# Monitoring the Evolution of Relative Product Populations at Early Times during a Photochemical Reaction

**DOI:** 10.1021/jacs.3c13046

**Published:** 2024-02-06

**Authors:** Joao Pedro Figueira Nunes, Lea Maria Ibele, Shashank Pathak, Andrew R. Attar, Surjendu Bhattacharyya, Rebecca Boll, Kurtis Borne, Martin Centurion, Benjamin Erk, Ming-Fu Lin, Ruaridh J. G. Forbes, Nathan Goff, Christopher S. Hansen, Matthias Hoffmann, David M. P. Holland, Rebecca A. Ingle, Duan Luo, Sri Bhavya Muvva, Alexander H. Reid, Arnaud Rouzée, Artem Rudenko, Sajib Kumar Saha, Xiaozhe Shen, Anbu Selvam Venkatachalam, Xijie Wang, Matt R. Ware, Stephen P. Weathersby, Kyle Wilkin, Thomas J. A. Wolf, Yanwei Xiong, Jie Yang, Michael N. R. Ashfold, Daniel Rolles, Basile F. E. Curchod

**Affiliations:** † University of Nebraska−Lincoln, Lincoln, Nebraska 68588, United States; ‡ CNRS, Institut de Chimie Physique UMR8000, Université Paris-Saclay, Orsay, 9140, France; § J.R. Macdonald Laboratory, Physics Department, Kansas State University, Manhattan, Kansas 66506, United States; ∥ SLAC National Accelerator Laboratory, Menlo Park, California 94025, United States; ⊥ European XFEL, Schenefeld, 22869, Germany; ▽ Deutsches Elektronen Synchrotron DESY, Hamburg, 22607, Germany; 7 Brown University, Providence, Rhode Island 02912, United States; 8 School of Chemistry, University of New South Wales, Sydney, NSW 2052, Australia; 9 Daresbury Laboratory, Warrington, WA4 4AD, U.K.; 10 Department of Chemistry, University College London, London, WC1H 0AJ, U.K.; 11 Max Born Institute, Berlin, 12489, Germany; 12 Stanford PULSE Institute, SLAC National Accelerator Laboratory, Menlo Park, California 94025, United States; 13 School of Chemistry, University of Bristol, Bristol, BS8 1TS, U.K.

## Abstract

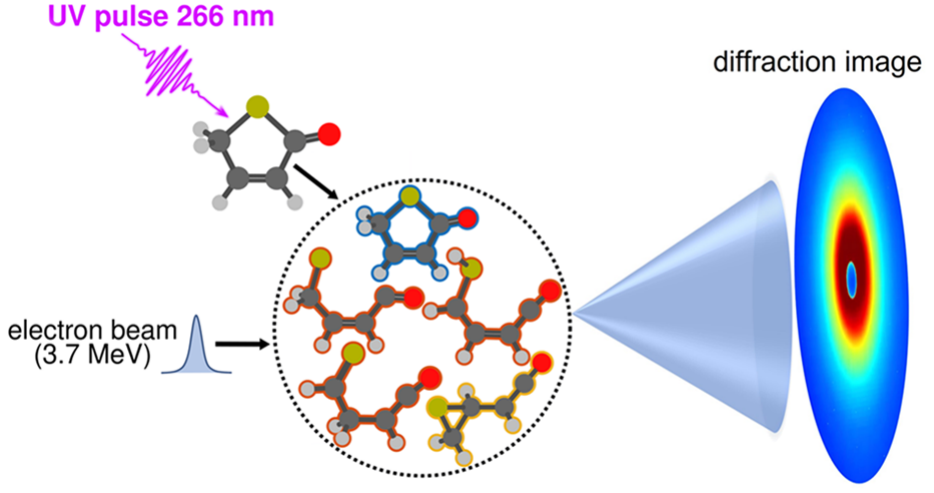

Identifying multiple rival reaction products and transient species formed during ultrafast photochemical reactions and determining their time-evolving relative populations are key steps toward understanding and predicting photochemical outcomes. Yet, most contemporary ultrafast studies struggle with clearly identifying and quantifying competing molecular structures/species among the emerging reaction products. Here, we show that mega-electronvolt ultrafast electron diffraction in combination with *ab initio* molecular dynamics calculations offer a powerful route to determining *time-resolved* populations of the various isomeric products formed after UV (266 nm) excitation of the five-membered heterocyclic molecule 2(5*H*)-thiophenone. This strategy provides experimental validation of the predicted high (∼50%) yield of an episulfide isomer containing a strained three-membered ring within ∼1 ps of photoexcitation and highlights the rapidity of interconversion between the rival highly vibrationally excited photoproducts in their ground electronic state.

## Introduction

Photochemistry addresses the consequences of molecules interacting with light. The field includes studies of the transformations a molecule can undergo following electronic excitation by absorbing an ultraviolet (UV) or visible photon. The desire to understand the formation mechanisms of products in photochemical processes—the photoproducts—has helped stimulate the development of a plethora of time-resolved spectroscopic and theoretical tools for investigating the dynamics of excited-state molecules.^1−8^

Some of these techniques are particularly suitable for probing ultrafast processes that occur in the excited electronic state(s) populated by photoexcitation. Such studies can provide rich information about the dynamics and time scales of photoinduced product formation. This should come as no surprise given that one of the founding tenets of photochemistry is that products are formed as a result of photoinduced changes in the electronic configuration of a molecule (i*.*e., products arise from excited electronic states).

More generally, however, photoproducts are formed after the photoexcited parent molecule has returned to its ground electronic state. The internal (vibrational) energy will almost certainly be dynamically determined (i.e., nonstatistically distributed among the normal modes) when the molecule first reappears in the ground state. Such an idea is not new—either in the context of photodissociations^9−12^ or of prototypical organic reactions occurring on the ground-state potential energy surface (PES)^13,14^—but definitive illustrations of dynamical effects determining the population and/or internal energy distributions of ground-state species formed by nonadiabatic transitions from higher electronic states (e.g., different ground-state isomers or eventual dissociation products) remain rare. The anharmonicity of the ground-state potential will encourage intramolecular vibrational redistribution (IVR), and the nascent vibrational energy distribution will evolve toward a more statistical microcanonical distribution over all internal modes. But, in the absence of collisions, as is the case in a low-pressure gas phase sample, the molecule cannot dissipate this energy. Any full understanding of photoinduced reaction dynamics in such cases thus also requires techniques that inform on the time scales of this vibrational energy flow and how (or whether) this evolving distribution of vibrational energy affects photoproduct formation. Deciphering the formation of photoproducts in the ground electronic state following a nonradiative decay requires a robust strategy to monitor the respective photoproduct populations in *real time*.

Time-resolved photoelectron spectroscopy (TRPES) is one technique that is very well suited to following the early time dynamics of photoexcited molecular systems.^15−21^ TRPES is, however, challenged both by the high photon energies (short wavelengths) required to probe molecules in their ground states and by the potential lack of selectivity when it comes to distinguishing photoproducts with similar electronic structures. Intensive theoretical calculations are often required to interpret experimental TRPES signals and extract information on the photoproducts, as in our recent ultrafast TRPES studies of the UV-photoinduced ring-opening of 2(5*H*)-thiophenone.^22^

**Scheme 1:**
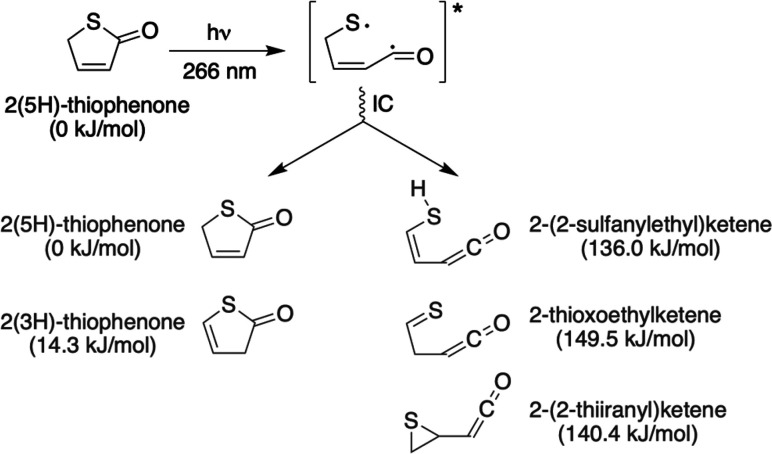
Photochemistry of 2(5*H*)-Thiophenone upon Excitation at 266 nm Ring-opening takes place in the excited electronic states leading to the formation of a biradical, which undergoes internal conversion (IC) to the ground electronic state where a range of possible photoproducts (dark gray) can be formed with high internal energies. Ring closure can take place, reforming the parent 2(5*H*)-thiophenone molecule or, potentially, 2(3*H*)-thiophenone. Alternatively, different ketenes can be formed, including the episulfide (2-(2-thiiranyl)ketene). The relative electronic energies for key structures are given between parentheses (calculated at the CCSD(T)-F12/cc-pVDZ-F12//MP2/6-311+G** level of theory).

The sulfur-containing five-member heterocycle 2(5*H*)-thiophenone displays a prototypical photochemical response upon UV light absorption^23,24^ (Scheme 1): a fast ring-opening process wherein one C–S bond breaks to form a ring-opened (acyclic) form and triggers an ultrafast decay toward the ground (S_0_) electronic state, which is accessed within 300 fs.^22^ The full photochemistry of 2(5*H*)-thiophenone involves much more than just this simple primary bond fission process, however. Further intramolecular rearrangements within the vibrationally excited ground-state species could potentially lead to reformation of a thiophenone (the 2(5*H*)- or 2(3*H*)-isomers) and/or isomerization to various ketenes. Among these ketenes, an exotic episulfide species, 2-(2-thiiranyl)ketene, involving an S-containing three-membered ring, has been predicted to dominate at early times following the nonradiative deexcitation (Scheme 1).^22,25,26^

**Figure 1:**
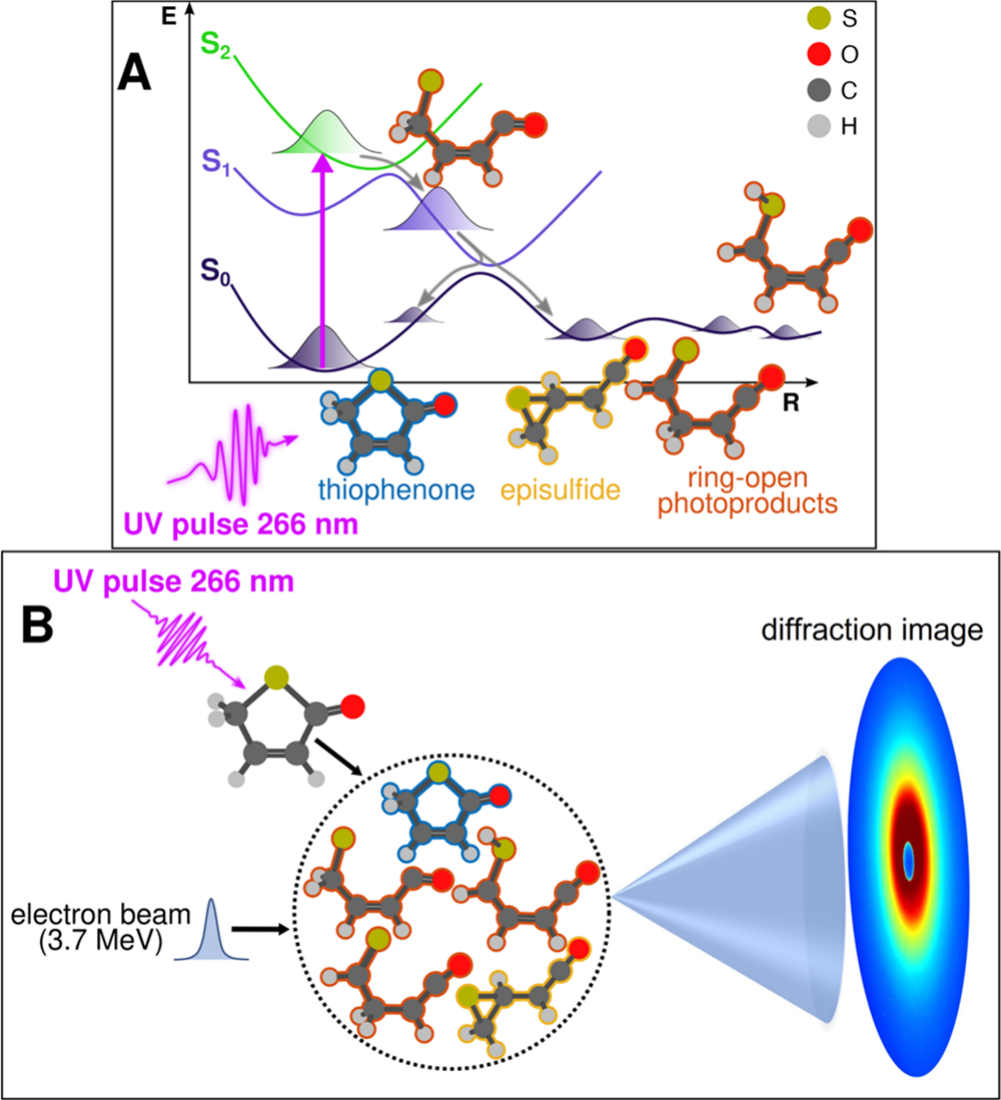
Schematic representation of the photochemistry of 2(5*H*)-thiophenone and the UED experiment. (A) Summary of the 2(5*H*)-thiophenone photochemistry following irradiation at 266 nm. Excitation to the second excited singlet electronic state (S_2_, green potential energy curve, plotted as a function of a generalized reaction coordinate **R**) results in immediate C–S bond extension (ring opening) and ultrafast nonradiative decay via the S_1_ (blue) to the ground (S_0_, dark purple) electronic states. The population of photoexcited 2(5*H*)-thiophenone molecules fully returns to the S_0_ state within 300 fs, whereupon the athermal dynamics drives formation of different families of photoproducts (presented in Scheme 1)—all with a substantial internal energy. (B) Schematic of the experimental setup. An ultrashort duration UV (266 nm) pulse photoexcites an ensemble of gas-phase 2(5*H*)-thiophenone molecules inside the UED target chamber, which is interrogated by an incident 3.7 MeV electron beam, yielding a diffraction pattern in reciprocal space that is recorded on a position-sensitive detector.

The formation of a three-membered ring from a five-membered cyclic species following the absorption of a 266 nm photon, with 4.65 eV of energy, might appear to challenge chemical intuition. Our recent TRPES studies using suitably short probe wavelengths^22^ succeeded in resolving the ultrafast nonradiative decay of 2(5*H*)-thiophenone from its second excited singlet electronic state (S_2_, with *n*(S)π* character in the Franck–Condon region) following photoexcitation at 266 nm (Figure 1A) and in revealing the formation of acyclic photoproducts. However, an unambiguous identification of the various products was limited by their very similar low-energy ionization potentials—all of which are associated with removing an electron from a relatively unperturbed sulfur lone pair—thus preventing definitive identification of the predicted episulfide.^22,25^

Here, we demonstrate that the sub-picosecond time resolution offered by the mega-electronvolt ultrafast electron diffraction (MeV-UED) at the SLAC National Accelerator Laboratory,^3,27−30^ again working hand in hand with theoretical chemistry, offers a very powerful complement, capable of identifying and distinguishing different product families following UV photoinduced ring-opening of 2(5*H*)-thiophenone and tracking the evolving photoproduct populations. The 2(5*H*)-thiophenone molecule is first excited with a UV pulse (266 nm) and then probed at different time delays with an electron beam at 3.7 MeV (Figure 1B). Analyzing the diffraction pattern of the electron beam provides a snapshot of the molecular geometries present at specific time delays following the UV pump pulse. The complementary combination of *ab initio* nonadiabatic and adiabatic Born–Oppenheimer molecular dynamics simulations allows calculation of photoproduct-family-specific basis functions, which are used to decompose the measured MeV-UED signals and determine the time-dependent relative populations of the various photoproducts.

## Methods

### Experimental Procedures

The experiment was performed at the MeV UED facility at the SLAC National Accelerator Laboratory. The experimental setup (see Figure S1 in the Supporting Information (SI)) is described in detail elsewhere.^31^ We note that earlier UED experiments highlighted the potential of this technique for tracking transient molecular structures.^32−34^ Briefly, the output of an 800 nm Ti:sapphire laser was split into two beams, each of which was frequency-tripled to generate femtosecond UV pulses. One of the UV beams was used as a “pump” to excite the 2(5*H*)-thiophenone molecules, while the other was used to generate ultrashort electron pulses by irradiating the photocathode of a radio frequency gun. The electron bunches of <150 fs (full width half-maximum, fwhm) duration^31^ containing ∼10^4^ electrons were accelerated to 3.7 MeV and focused to a spot size of 200 μm fwhm in the interaction region of the gas-phase experimental chamber, where they interacted with the 2(5*H*)-thiophenone molecules that were delivered into the high-vacuum interaction region in a continuous flow gas cell 3 mm in length with 550 μm openings. 2(5*H*)-Thiophenone (98% purity) was purchased from abcr GmbH and used without further purification. Since 2(5*H*)-thiophenone has a low vapor pressure (<1 Torr) at room temperature, the sample reservoir and delivery assembly were heated to 60 °C. The pump pulses (15 μJ, 266 nm, ∼67 fs (fwhm) in duration) were focused into the interaction chamber to a diameter of 240 μm fwhm and were overlapped with the electron pulses at a 1° crossing angle using a holey mirror. Both the pump laser pulses and the MeV electron pulses were delivered at a repetition rate of 360 Hz. The scattered electrons were detected on a P43 phosphor screen, which was imaged by an Andor iXon Ultra 888 EMCCD camera. Diffraction data were acquired at 37 unique time delays between −3 and 10 ps (Figure S2). Each time delay was visited a single time per scan, and the order in which delays were visited was randomized between scans to minimize systematic errors. In each scan, diffraction data were acquired for 10 s (3600 electron shots) at each time delay. A total of 178 scans were acquired, yielding a total integration time per time delay of ∼30 min. The instrument response function (IRF) of the MeV UED apparatus^31^ as used in the present pump–probe configuration is estimated to be 230 fs (fwhm, see Figure S9A in the SI). Additional data validating the assumption that the photochemical findings reported herein are the result of single UV photon excitation processes are presented in the SI (Figures S11 and S12).

### Nonadiabatic and *ab Initio* Molecular Dynamics

The nonadiabatic molecular dynamics (NAMD) following instantaneous photoexcitation of 2(5*H*)-thiophenone were simulated using Tully’s fewest-switches trajectory surface hopping^35^ method with the SHARC program package^36,37^ as described in ref (22). Forty three initial conditions, sampled from a Wigner distribution for uncoupled harmonic oscillators, were initialized in the bright S_2_ state. The electronic structure was described at the SA(4)-CASSCF(10/8)/6-31G* level of theory using the Molpro 2012 program package.^38,39^ This level of theory was thoroughly benchmarked in ref (22) against XMS-CASPT2. The energy-based decoherence correction scheme was employed.^40,41^ After a surface hop, the kinetic energy was rescaled isotropically. A nuclear time step of 0.5 fs was used for all of the NAMD trajectories. During the excited-state dynamics, the total energy along each trajectory was strictly conserved. Upon deactivation to the ground state, each NAMD trajectory was propagated further until it left the region of strong nonadiabaticity between the ground and first excited electronic state. At this point, the NAMD trajectory was stopped and (ground-state) *ab initio* Born–Oppenheimer molecular dynamics (BOMD) initiated, using the last step of the NAMD trajectory to define the initial conditions. The BOMD trajectories were propagated on the ground electronic state using unrestricted DFT employing the PBE0 exchange/correlation functional^42^ and the 6-31G* basis set, with the GPU-accelerated software TeraChem.^43,44^ The BOMD simulations were carried out until a total simulation time (the sum of NAMD and BOMD simulation times) of 2 ps, using a reduced time step of 0.1 fs. A benchmark of this strategy as well as a discussion of the (minimal) influence of triplet states and intersystem crossing is presented in the SI of ref (22).

The geometries captured by the (NA+BO)MD simulations were classified into photoproduct categories according to the decision trees shown in Figure S4 in the SI. This classification relies on the identification of characteristic atomic connectivities using bond lengths or angles. The decision tree for classification I, shown in Figure S4A, allows the identification of all unique photoproducts captured in the (NA+BO)MD simulations and is inspired by the decision tree reported in ref (22). The scattering signals produced by the photoproducts identified under classification I, depicted in Figure S5A in the SI, show that ring-opened geometries produce very similar signatures, which cannot be distinguished given the uncertainties within the experimental signal. Therefore, in classification II, shown in Figure S4B, the ring-opened photoproducts (the excited ring-opened form of 2(5*H*)-thiophenone and all the acyclic ketene products, see Scheme 1) were grouped under a single classification, *ring-opened*, to ensure that the scattering signatures of all classified photoproducts can be unambiguously distinguished in the experimental signal. The scattering signatures of photoproducts identified under classification II are shown in Figure S5B (SI).

### Analysis of Electron Diffraction Data and Determination of Photoproduct Branching Ratios

Full details of the electron diffraction data analysis and the routes to extracting photoproduct branching ratios are described in the SI. Briefly, the two-dimensional diffraction patterns recorded at the EMCCD detector were processed into one-dimensional scattering intensity curves, *I*(*s*), where *s* is the momentum transfer vector, which were then decomposed into atomic and molecular scattering contributions. The diffraction data were then converted to modified scattering intensities, *sM*(*s*), to enhance the oscillations in the molecular scattering term and suppress the rapid drop in scattering intensity as a function of *s* imparted by the *s*^–2^ scaling in the elastic scattering amplitude (see SI). Approximating the *sM*(*s*) curve as a sum of sine waves (arising from all internuclear distances in the target molecule) allowed its decomposition into a pair-distribution function (PDF) of all contributing interatomic distances.

**Figure 2:**
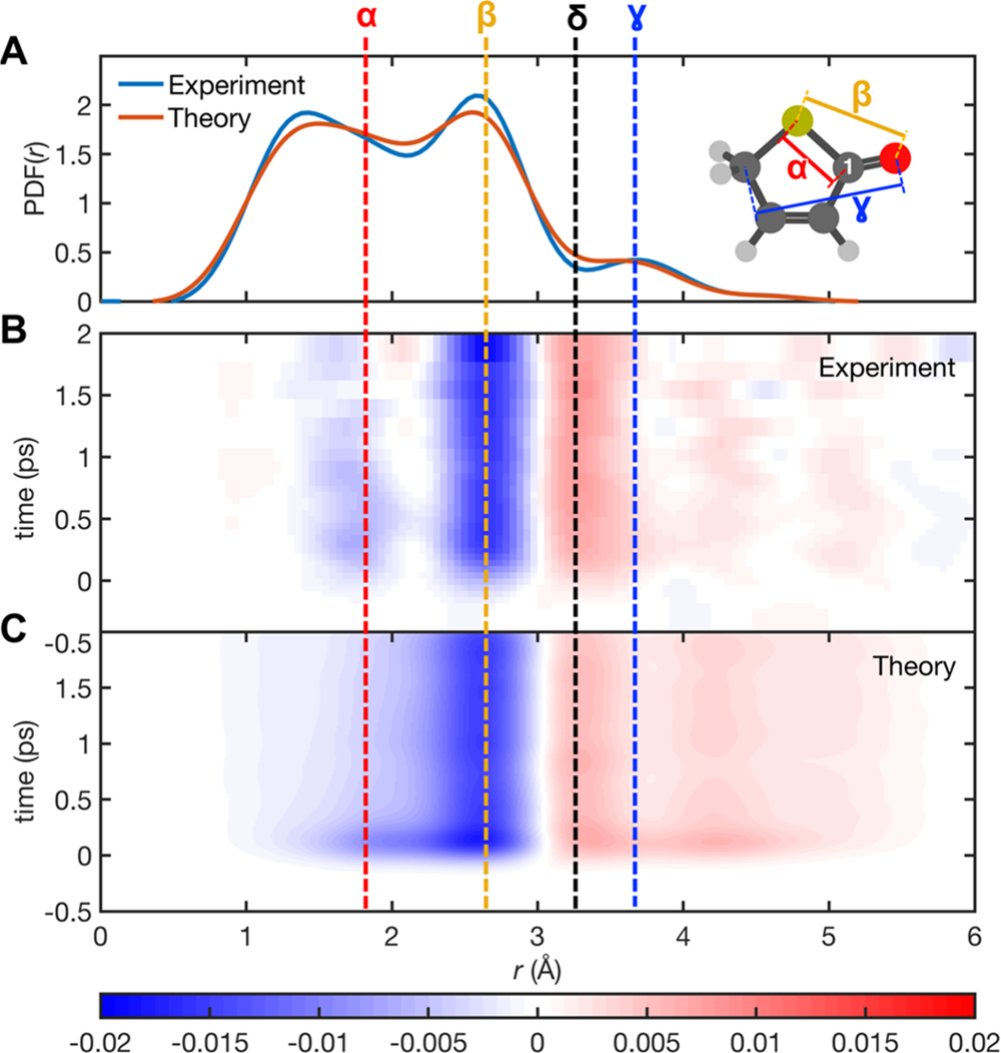
Comparisons between the experimentally derived and theoretically predicted PDFs and the ΔPDF(*r*,*t*)) maps. (A) Experimentally derived and theoretically predicted steady-state PDFs for 2(5*H*)-thiophenone. See SI for the full assignment of the steady-state PDFs for 2(5*H*)-thiophenone (Figure S10). (B and C) False-color plots of the experimental and theoretical ΔPDF are shown as a function of pump–probe time delay. The latter summarizes the dynamics observed for a swarm of 43 trajectories obtained from trajectory surface hopping simulations (SA4-CASSCF(10/8)/6-31G*) for the NAMD from S_2_ to S_0_, further continued in S_0_ with BOMD (UDFT/PBE0/6-31G*). The signal shown in (C) was reconstructed from the trajectories using the independent atom model, convolved with a 230 fs full width at half-maximum (fwhm) Gaussian function to approximate the IRF. The separations associated with the three strongest features of the static PDF, α (red), β (yellow), and γ (blue), and an obvious photoinduced feature, δ (black), are highlighted using vertical dashed lines. The interatomic spacings that contribute most to the α, β, and γ features are shown by the color-coded tie-lines overlaid on the optimized structure of ground-state 2(5*H*)-thiophenone, which also identifies the carbonyl C atom (C1) that features later in the narrative.

Analysis of the time-resolved experimental UED data was based on the difference-diffraction method,^45^ wherein the fractional change signal, Δ*I*/*I*(*s*,*t*) is defined as
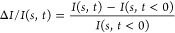
1where *I*(*s*,*t*<0) is the reference diffraction signal taken before the arrival of the pump pulse and *I*(*s*,*t*) is the diffraction intensity recorded at pump–probe delay *t*. The experimental time-dependent difference pair distribution functions, ΔPDF(*r*,*t*), shown in Figure 2 were calculated by applying the sine-transform of the time-dependent difference-modified scattering curves.

The theoretical static PDF(*r*) and time-dependent Δ*I*/*I*(*s*,*t*) and ΔPDF(*r*,*t*) signals reported here were calculated within the independent atom model^46,47^ (IAM) using the nuclear configurations captured along the 43 (NA+BO)MD trajectories. The relative populations for the photoproducts were retrieved directly from the UED signal by fitting a linear combination of basis functions (for thiophenone, acyclic (ring-opened) ketene, and episulfide products) selected to reflect the average scattering signatures of these three photoproduct families to the experimental difference-diffraction signal, Δ*I*/*I*(*s*,*t*). These photoproduct basis functions were obtained by averaging the theoretical Δ*I*/*I*(*s*,*t*) signals for thiophenone, ring-opened, and episulfide products in the range 1 ≤ *t* ≤ 2 ps. This approach allows the determination of the relative abundances of these three basis functions and their evolution across the experimental time window. These returned relative abundances correspond to relative product populations provided the IAM is valid, i.e., that each isomer of a common ground-state species (neutral C_4_H_4_SO in this case) shows the same elastic diffraction intensity.

## Results and Discussion

### Steady-State and Time-Resolved Atomic Pair Distribution Functions

The ΔPDF(*r*,*t*) maps obtained from the UED measurements following 266 nm photoexcitation of 2(5*H*)-thiophenone at pump–probe time delays out to *t* = 2 ps (Figure 2B) reveal an obvious decrease in the most intense feature in the parent static PDF (Figure 2A). The theoretical ΔPDF(*r*,*t*) map (Figure 2C) calculated from a swarm of combined nonadiabatic and ground-state dynamics trajectories (see Methods) is in excellent agreement with the experimental data, reproducing all of the main features. The experimental and theoretical ΔPDF(*r*,*t*) maps both show an obvious photoinduced feature (δ, dashed black line in Figure 2), but the α, β, and γ features show seemingly different responses to photoexcitation. The first two, which contain substantial contributions from, respectively, the ring-closed parent C···S (including the C1···S bond that breaks) and S···O separations, both show predictable decreases when some of the parent population is converted to photoproducts, but the γ feature shows no similar decrease. As we now show, these differences and the entire ΔPDF(*r*,*t*) maps are completely understandable by recognizing that parent depletion (by photoexcitation) is quickly followed by photoproduct formation once the excited molecules reappear in the S_0_ state with athermal population and internal energy distributions. The feature labeled δ in the ΔPDF(*r*,*t*) map already hints at the formation of the predicted episulfide, but additional analysis is required to make this assignment unambiguous and to quantify the episulfide yield.

### Monitoring the Appearance of the Episulfide Molecule

**Figure 3:**
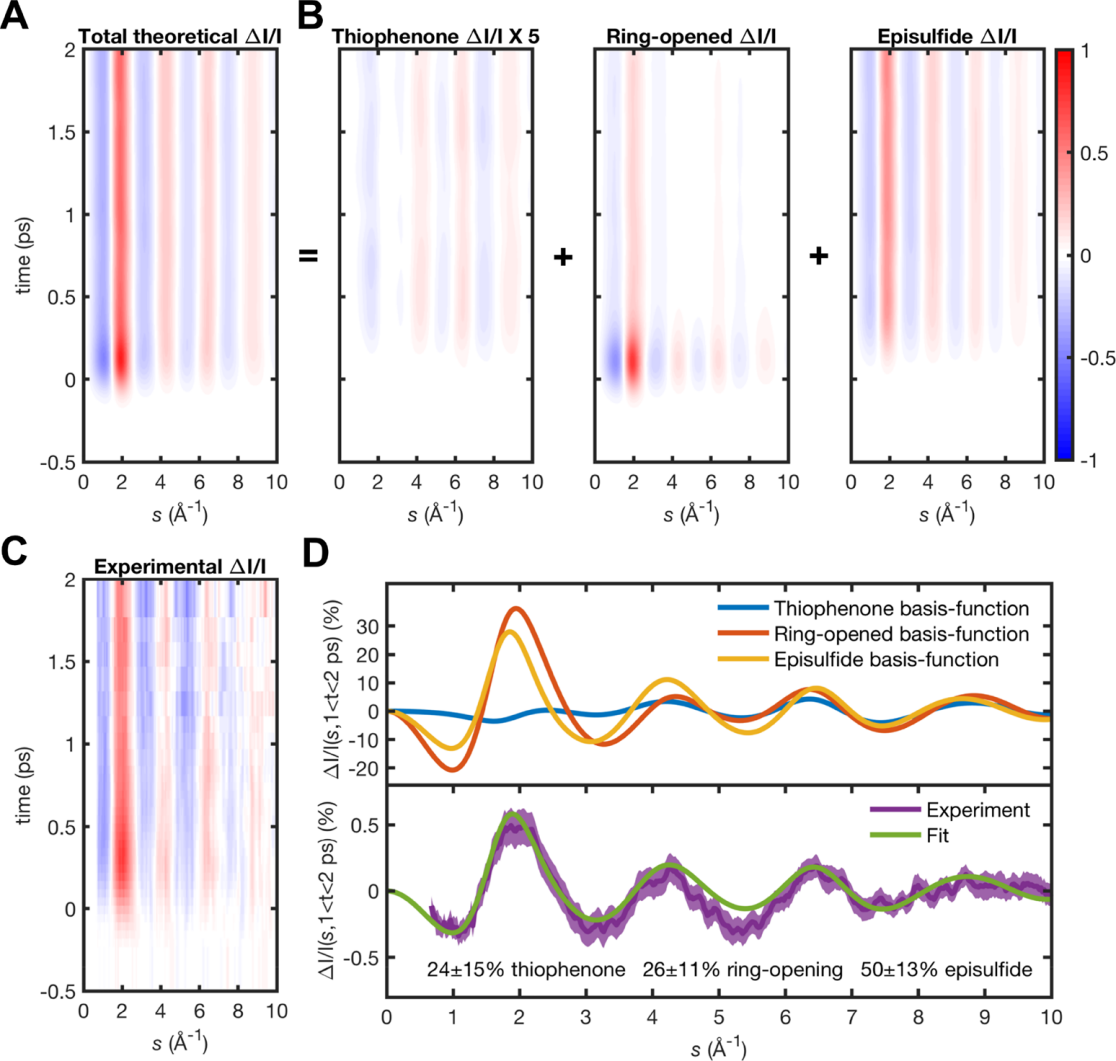
Contributions of the different photoproducts to the Δ*I*/*I* UED signal. (A and B) Theoretical total Δ*I*/*I*(*s*,*t*) map and the Δ*I*/*I*(*s*,*t*) contributions from each family of photoproducts, each of which has been scaled by the experimental excitation percentage (∼3%) and convolved with a 230 fs fwhm Gaussian function to approximate the IRF. (C) False-color plot of the measured Δ*I*/*I*(*s*,*t*) vs *s*. (D) Upper panel: scattering Δ*I*/*I*(*s*,*t*) vs *s* signatures for 2(5*H*)-thiophenone, ring-opened, and episulfide photoproducts, based on the average of the theoretical Δ*I*/*I*(*s*,*t*) signals of classified structures extracted from trajectories in the time interval 1–2 ps; lower panel: comparison between the experimental Δ*I*/*I*(*s*,*t*) signal and the fit obtained by using the theoretical signature for each photoproduct depicted in the upper panel and the stated product branching ratios.

To pinpoint the presence of the episulfide product, it proves more revealing to work in terms of Δ*I*/*I*(*s*,*t*) and *s*, where Δ*I* is the photoinduced change in radially averaged diffraction intensity relative to that from the unexcited sample (i.e., that recorded when the UED probe pulse preceded the 266 nm pump pulse) and *s* is the momentum transfer vector (see Methods and SI for additional information). Figures 3C and 3A show, respectively, the Δ*I*/*I*(*s*,*t*) maps measured by UED and calculated from the structures returned by the *ab initio* nonadiabatic and ground-state dynamics simulations for the time delay range of −0.5 ≤ *t* ≤ +2.0 ps.

**Figure 4:**
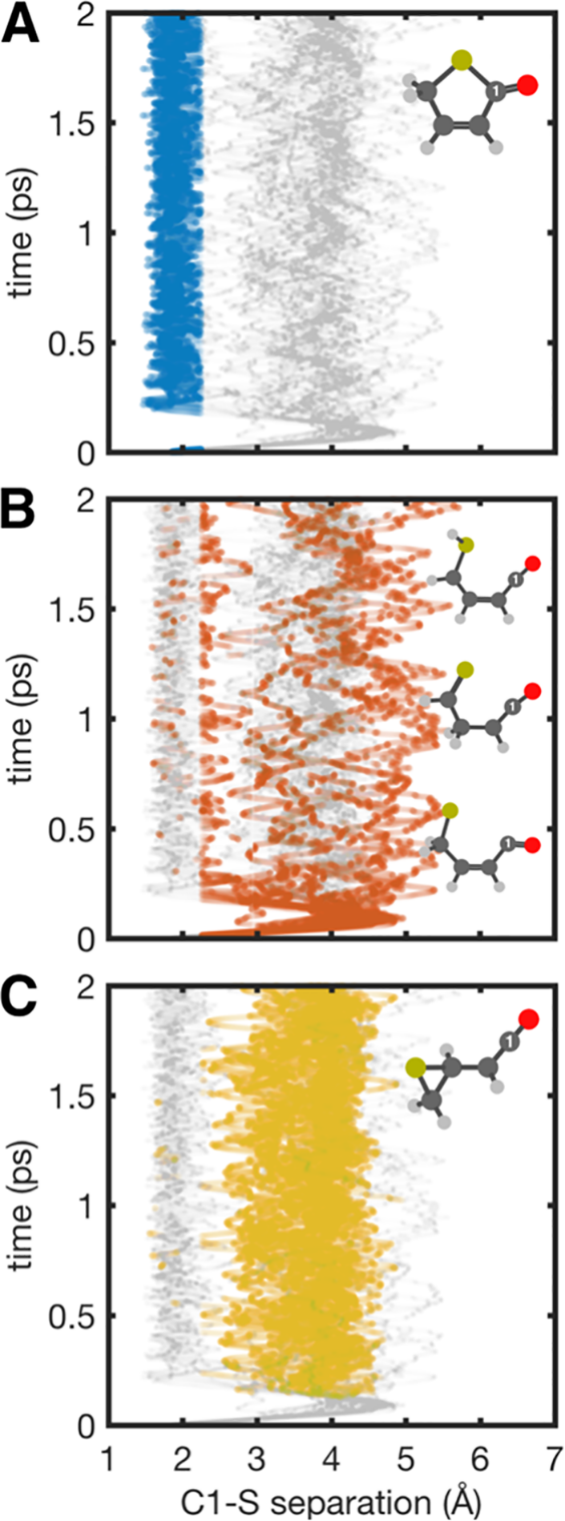
Calculated time-varying C1···S separation for the full swarm of trajectories (gray) and, respectively, 2(5*H*)-thiophenone (A, blue), ring-opened molecules (B, orange), and episulfide (C, yellow).

We first focus on the theoretically predicted Δ*I*/*I*(*s*,*t*) map (Figure 3A). Each combined (NA+BO)MD simulation returns nuclear coordinates (i.e., a structure) at each time step, from which the Δ*I*/*I*(*s*) pattern can be calculated (see Methods and the SI). Figure 3A shows the sum of all such contributions at each time step. Each instantaneous structure in each simulation can also be assigned to one of three *families* of photoproducts: ring-closed (designating return as an internally excited ring-closed thiophenone); ring-opened (i.e., excited-state biradicals at very early time delays and the acyclic thioenol or thioaldehyde isomers after reversion to the S_0_ state); and the proposed episulfide (see Methods and Figure S4 in the SI for details on the classification). We stress at this point that the simulations show zero formation of the ring-closed 2(3*H*)-thiophenone within the 2 ps dynamics presented here; ring-closing in the S_0_ state results solely in reformation of the parent 2(5*H*)-isomer. Figures 4A–4C highlight the very different amplitudes of the C1···S separations associated with instantaneous structures assigned to each photoproduct family. Representative structures from each photoproduct family are displayed in these panels. The total Δ*I*/*I*(*s*,*t*) map (Figure 3A) is simply the sum of the photoproduct-specific Δ*I*/*I*(*s*,*t*) maps shown in Figure 3B, weighted according to the relative populations of the photoproduct families.

Inspection of the photoproduct-specific and total Δ*I*/*I*(*s*,*t*) maps (Figures 3A–3C) reveals that the ring-opened structures make substantial contributions immediately after photoexcitation, consistent with the initial 2(5*H*)-thiophenone ring-opening (see the C1···S separation plot in Figure 4B). They also show that, within the time frame probed, the respective diffraction signatures are largely insensitive to time once all molecules have reverted to the S_0_ state (i.e., after ∼0.5 ps; see the SI for an extended discussion). Figure 3D (upper panel) shows the “average” Δ*I*/*I*(*s*) signatures derived from the (NA+BO)MD simulations for each photoproduct family during the period +1.0 ≤ *t* ≤ +2.0 ps, which we can use as time-independent basis functions (see Figure S6 in the SI) to decompose the total Δ*I*/*I*(*s*,*t*) maps. This is a key aspect of the present analysis: using these basis functions to fit the experimental Δ*I*/*I*(*s*,*t*) signal integrated over the period +1.0 ≤ *t* ≤ +2.0 ps provides clear evidence that the episulfide is formed upon UV irradiation of 2(5*H*)-thiophenone (Figure 3D, lower panel).

The best reproduction of the features observed in the raw data between 1.0 ≤ *s* ≤ 4.0 Å^–1^ (the region of reciprocal space that offers the best signal-to-noise ratio) is obtained by including a substantial contribution from the basis function for episulfide (see the SI for further validations of this procedure and the potential limitations of treating the data with a high band-pass filter). Further, the distinctive nature of the UED signal allows estimation of the relative population of the episulfide photoproduct: 50 ± 13% over the 1–2 ps time window. Strictly, this latter decomposition returns the time-dependent fractional contribution of each basis signature to the experimental Δ*I*/*I*(*s*,*t*) data. These ratios will correspond to photoproduct population ratios provided that the independent atom model is valid, i.e., that each isomer of a common ground-state species (here the neutral C_4_H_4_SO species) shows the same elastic diffraction intensity.^45,48−50^

The present study also serves to emphasize the importance of extracting such theoretical basis functions from geometries representative of the athermal distribution of the photoproducts, *not* simply from their ground-state optimized geometries or from some assumed thermalized distribution. Figure S15 in the SI illustrates the very different static PDF(*r*) and *sM*(*s*) profiles predicted for such distributions of 2(5*H*)-thiophenone molecules. The quantitative analysis of the UED signal provided here requires great care in the processing of the data, as is also detailed in the SI (Figures S6–S8). Overall, the combination of UED and theory proves episulfide formation within a few hundreds of fs following 266 nm photoexcitation of 2(5*H*)-thiophenone.

### Extracting Time-Resolved Photoproduct Populations

The foregoing analysis returned a relative population of episulfide photoproducts in the +1.0 ≤ *t* ≤ +2.0 ps time window, but the fitting strategy combining theory and experimental UED signals allows us to go a step further: performing an analogous fit to UED data recorded at each experimental time delay reveals the *time dependence* of the different photoproduct populations.

**Figure 5:**
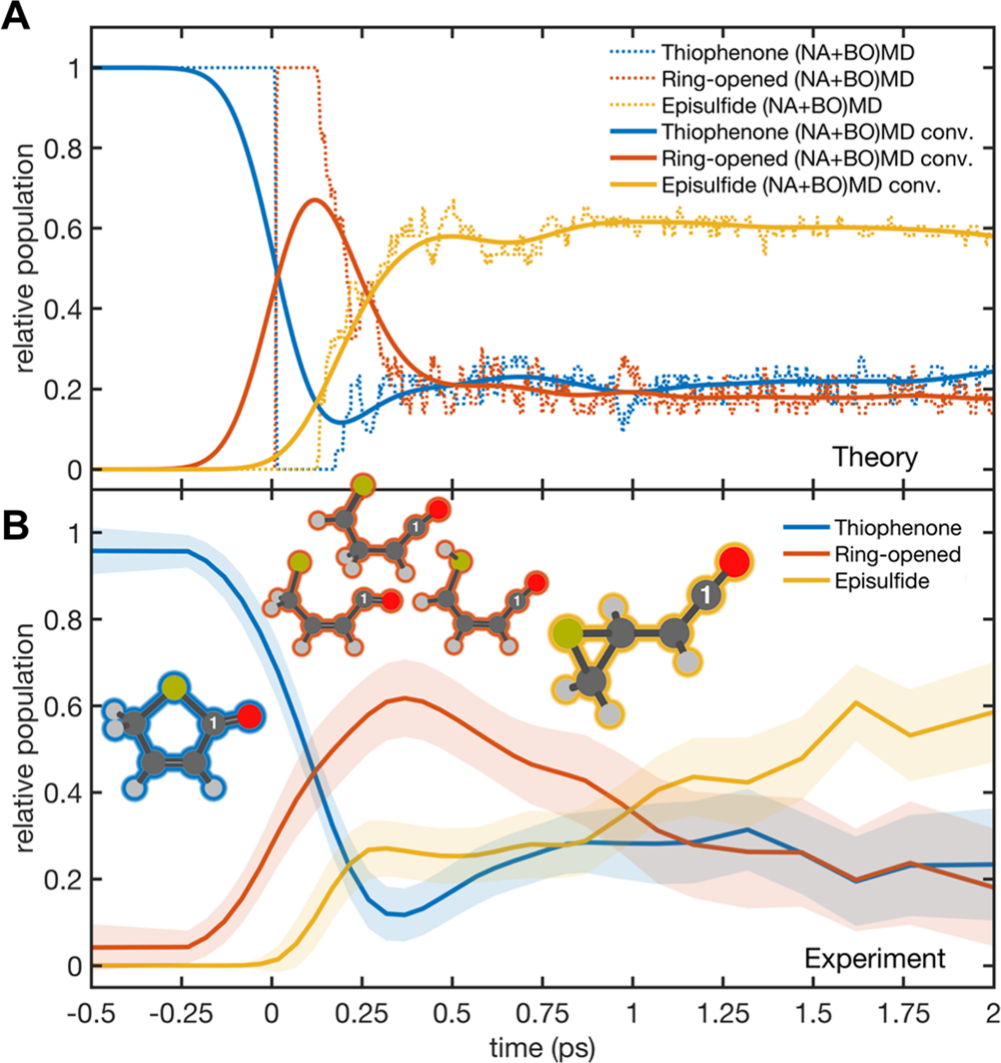
Time-resolved relative populations for the three families of photoproducts. (A) Time-resolved relative populations of product isomers obtained directly from the combined (NA+BO)MD simulations: 2(5*H*)-thiophenone (blue), ring-open forms (orange), and episulfide (yellow). The classification used to unravel these populations is discussed in the SI. The dotted lines show the populations as obtained from the dynamic calculations, while the solid lines are the same data convoluted by a function representing the IRF. (B) Time-dependent relative populations of products extracted from the experimental UED signal.

The (NA+BO)MD-predicted, time-dependent relative populations are shown by dotted curves in Figure 5A and again as solid curves after convolving with a 230 fs fwhm Gaussian function used as an approximate IRF. Figure 5B shows the results of a similar decomposition of the UED-derived total Δ*I*/*I*(*s*,*t*) map shown in Figure 3C. Experiment and theory are in excellent agreement with regard to the ultrafast disappearance of the parent 2(5*H*)-thiophenone molecule (blue traces in Figure 5) and the rise of a ring-opened structure (orange traces) following photoexcitation, in this case a biradical photoproduct emerging from the ring-opening of 2(5*H*)-thiophenone. Theory and experiment further agree on the timing of the birth of episulfide (yellow traces in Figure 5), taking place ∼200 fs after the appearance of the ring-opened photoproduct.

While theory and experiment agree on the photoproduct relative populations at 2 ps, theory predicts a faster growth of the episulfide population. This difference can be explained by the way the NAMD is initialized at *t* = 0, where the ground-state molecular wave function is perfectly projected onto S_2_, mimicking the photoexcitation that would be obtained with a δ-pulse.^51^ This initialization represents the formation of a perfectly coherent nuclear wavepacket in S_2_—a molecular state that only approximates the experimentally induced molecular state obtained by the progressive population of the excited state with a 67 fs laser pulse. We also note that the (NA+BO)MD employs a swarm of classical, independent trajectories whose evolution tends to appear more (classically) coherent.

Notwithstanding the foregoing caveat, the level of agreement between the experiment and theory is very satisfying. Both show that photoexcitation to the S_2_ state drives immediate C1–S bond extension (manifested as loss of 2(5*H*)-thiophenone and a decline in the blue trace in Figure 5), leading to ring-opening (the early time rise in the orange trace in Figure 5), which enables reversion to the S_0_ state.^22^ These conclusions are entirely consistent with those of the previously reported TRPES study of UV photoexcited 2(5*H*)-thiophenone molecules,^22^ with the critical difference that the UED measurements presented here additionally allow us to (i) identify the episulfide unequivocally and (ii) extract time-dependent relative populations for the different photoproduct families.

### Dynamics Govern the Product Population Distributions

The present UED measurements and complementary modeling also provide a wealth of hitherto inaccessible dynamical insights. Once back in the S_0_ state, the ring-opened biradical species either convert to ring-closed parent 2(5*H*)-thiophenone molecules (revealed by the rise after 250 fs in the blue trace in Figure 5) or form ring-opened and episulfide photoproducts (shown by, respectively, the orange and yellow traces in Figure 5). We reiterate that the molecular simulation shows that ring-closing results exclusively in reformation of the 2(5*H*)-parent isomer. As also noted above, the UED signal analysis cannot distinguish between the three acyclic (ring-opened) photoproducts, but information about the appearance of these molecules can be extracted from the (NA+BO)MD dynamics. The simulations (Figure S14) show ground-state 2-(2-sulfanethyl)ketene and 2-thioxoethylketene (Scheme 1) appearing within 100 fs of the photoexcitation process, with respective populations fluctuating around ∼10% of all photoproducts within 200 fs. The simulations also illustrate the rapidity (sub-picosecond time scale) of the interconversion between the different highly internally excited ring-opened photoproduct isomers by H atom transfer between adjacent heavy atoms. The decline of the ring-opened biradical population exhibits two time scales: an initial fast decline within 250 fs, followed by a further more gradual decline to below 10% of the total photoproduct population within the 2 ps of the present dynamics simulations (see the SI).

It is also important to note that energy and momentum conservation dictate that all these photoproducts must carry high levels of vibrational excitation, the distribution of which among the available normal modes will be determined by the dynamics of the initial bond fission. Such athermal early time nuclear motions are increasingly becoming recognized as a potential trigger for unexpected (i.e., nonstatistical) ground-state reactivity.^12,14,52−55^ The (NA+BO)MD simulations highlight the fluxional nature of these highly internally excited photoproducts, which interconvert between the various structural families on a sub-picosecond time scale (as can be seen from the all-trajectory C1···S separation vs time plots shown in Figure 4). Thus, it is important to appreciate that, though the UED (and (NA+BO)MD) data and their analyses provide product branching ratios, the photoproduct identities are not frozen by the end of the time window sampled in the present study. Any one photoproduct molecule carries more than sufficient internal energy to be able to interconvert among other photoproduct families.

Comparisons with the respective product populations for a canonical distribution of ground-state molecules maintained at a temperature corresponding to *E* ∼ 4.65 eV (*T* ∼ 2200 K) are also instructive. A simple Boltzmann calculation based on the respective ground-state electronic energies returns the population ratios thiophenone/ring-opened/episulfide ∼ 99.87:0.08:0.05%. Some 31% of the thiophenone species would be predicted to be in the form of the 2(3*H*)-isomer given the relative ground-state energies shown in Scheme 1. The observed predominance of episulfide photoproducts and the complete absence of 2(3*H*)-thiophenone ring-closed products returned by the simulations emphasize that the early time photoproduct population distributions are dynamically determined. As noted before, anharmonicity will encourage a gradual “thermalization” of internal energy among all normal modes of the “hot” photoproducts and an evolution toward a more statistical product population distribution over much longer time scales. However, as also noted previously,^22^ the total available internal energy is sufficient to enable unimolecular decay of the acyclic photoproducts (e*.*g., CO loss) at later times.

The agreement between theory and experiment confirms the exotic episulfide as a major photoproduct, with a relative population of 50 ± 13% at early times. Episulfide product formation explains the different photoinduced reductions in the features labeled α and β in Figure 2: the O···S separation in the episulfide (and the ring-opened photoproducts) is much larger than that in the parent 2(5*H*)-thiophenone, so all such transformations lead to a reduction in the peak labeled β. But the episulfide photoproducts contain C–S bond separations similar to those in 2(5*H*)-thiophenone. This, plus the fact that one C–S bond survives in the initial ring-opening, explains the lesser reduction in the intensity of the feature labeled α. Even the β peak is likely to be less reduced than might be expected based on the zero-order assumption that it reports just on the O···S separation, since the longer C···O separation in the C=C=O group within the photoproducts will also contribute to the elastic diffraction signal at *r* ∼ 2.5 Å. All photoproducts contain C···O pairs with similar separations to that in the 2(5*H*)-thiophenone precursor—accounting for the apparent insensitivity of the feature labeled γ to photoexcitation. Reference to Figure 4C shows that the C1···S separation in the episulfide product is a major contributor to the photoinduced feature labeled δ in Figure 2.

## Conclusion

This study illustrates how a combination of *ab initio* (nonadiabatic and adiabatic) molecular dynamics simulations and contemporary MeV-UED probe techniques allows (i) identification of the episulfide isomer following UV excitation of 2(5*H*)-thiophenone and (ii) determination of time-dependent relative populations of the different families of photoproduct isomers at early times following nonadiabatic coupling back to the ground-state potential. As noted at the outset, reversion to the ground electronic state is a common fate for molecules following photoexcitation. This demonstration study highlights the importance of dynamics in determining the relative populations of photoproducts and the rapid (sub-picosecond time scale) interconversion between the different highly internally excited acyclic photoproduct isomers enabled by transferring an H atom between neighboring heavy atoms. It points the way to a wealth of future studies designed to identify all major products from such photoinduced athermal ground-state chemistry and to determine their relative populations and how these evolve at early times—a spread of insights currently offered by few (if any) other ultrafast probe methods.

## Data Availability

The raw UED data set (15 mJ) and trajectories used in this work are available at the following link: doi.org/10.5281/zenodo.10045070.

## Supporting Information Available

Detailed description of the experimental setup, data processing, error estimation, full computational details, fitting procedures and benchmarking (PDF)
