# Supplementary material for: Monitoring the Evolution of Relative Product Populations at Early Times during a Photochemical Reaction
Source: J Am Chem Soc. 2024 Feb 6;146(6):4134–43. doi: 10.1021/jacs.3c13046 (PMC10870701; doi:10.1021/jacs.3c13046)
Supplement: Supplementary file 1 — ja3c13046_si_001.pdf [file ja3c13046_si_001.pdf]

Supporting Information for

Monitoring the evolution of relative product populations at early times during a photochemical reaction

Joao Pedro Figueira Nunes[†1], Lea Maria Ibele[†2], Shashank Pathak[†3], Andrew R. Attar[4=], Surjendu Bhattacharyya[3], Rebecca Boll[5], Kurtis Borne[3], Martin Centurion[1], Benjamin Erk[6], Ming-Fu Lin[4], Ruaridh J.G. Forbes[4], Nathan Goff[7], Christopher S. Hansen[8], Matthias Hoffmann[4], David M.P. Holland[9], Rebecca A. Ingle[10], Duan Luo[4], Sri Bhavya Muvva[1], Alexander H. Reid[4], Arnaud Rouzée[11], Artem Rudenko[3], Sajib Kumar Saha[1], Xiaozhe Shen[4], Anbu Selvam Venkatachalam[3], Xijie Wang[4], Matt R. Ware[4#], Stephen P. Weathersby[4], Kyle Wilkin[1], Thomas J.A. Wolf[4,12], Yanwei Xiong[1], Jie Yang[4,$], Michael N. R. Ashfold*[13], Daniel Rolles*[3], Basile F. E. Curchod*[13]

[1]University of Nebraska–Lincoln, Lincoln, NE, 68588, USA; [2]CNRS, Institut de Chimie Physique UMR8000, Université Paris-Saclay, Orsay, 9140, France; [3]J.R. Macdonald Laboratory, Physics Department, Kansas State University, Manhattan, KS, 66506, USA; [4]SLAC National Accelerator Laboratory, Menlo Park, CA, 94025, USA; [5]European XFEL, Schenefeld, 22869, Germany; [6]Deutsches Elektronen Synchrotron DESY, Hamburg, 22607, Germany; [7]Brown University, Providence, RI, 02912, USA; [8]School of Chemistry, University of New South Wales, Sydney, NSW, 2052 Australia; [9]Daresbury Laboratory, Warrington, WA4 4AD, UK; [10]Department of Chemistry, University College London, London, WC1H 0AJ, UK, [11]Max Born Institute, Berlin, 12489, Germany; [12]Stanford PULSE Institute, SLAC National Accelerator Laboratory, Menlo Park, CA, 94025, USA; [13]School of Chemistry, University of Bristol, Bristol, BS8 1TS, UK

[$]Present address: Center of Basic Molecular Science, Department of Chemistry, Tsinghua University, Beijing, 100084, China.
[#]Present address: SRI International, Boulder, CO, 80302, USA.
[=]Present address: Vescent Photonics, Golden, CO, 80401, USA.

†These authors contributed equally to this work.
*mike.ashfold@bristol.ac.uk; rolles@phys.ksu.edu; basile.curchod@bristol.ac.uk

Material and Methods

<u>*Experiment*</u>

Experimental data processing:

Two-dimensional diffraction patterns recorded at the EMCCD detector were processed into one-dimensional scattering intensity curves, $I(s)$, using the workflow listed below:

1. Outliers removal - Pixels with intensities 4 standard-deviations above the mean of identical pixels, *i.e.* same time delay and detector coordinate, were removed.
2. Data averaging - Diffraction patterns acquired at the same time delay were averaged together.
3. Background removal - An estimated background frame generated by linearly interpolating between the intensities of the four corners of the average image frame was subtracted from all diffraction image frames.
4. Detector mask - Masks were applied to all areas of the detector which do not image the phosphor screen and therefore do not contain scattering information. These areas include the edges of the image and the hole at the center of the detector, through which the transmitted electron beam passes.
5. Diffraction center assessment - The center of each diffraction pattern was determined by fitting circles to pixels with identical intensities (isolines).
6. Radial outlier removal - Pixels with intensities over 3 standard-deviations above the mean of pixels at the same radial distance from the diffraction center were removed.
7. Data reduction - Two-dimensional diffraction images frames were radially averaged around the diffraction pattern center to produce one-dimensional scattering intensity curves.
8. Data normalization - Scattering intensity curves were normalized based on the mean counts across the detected momentum transfer range: $0.67 < s < 12$ Å^{-1}.
9. Baseline subtraction - A power function ($a \times x^b$) is fitted to, and subtracted from, all one-dimensional scattering intensity curves.

Generating pair distribution functions from the experimental data:

Scattering intensities are measured at the detector as a function of the momentum transfer vector, s, defined as:

$$s = \frac{4\pi}{\lambda} sin(\frac{\theta}{2}) \qquad \text{(eq. S1)}$$

where λ is the de Broglie wavelength of the incident electrons and θ is the angle between the incident and scattered electrons. Under the independent atom model (IAM) approximation, the

total scattering intensity, $I(s)$, can be decomposed into molecular and atomic scattering contributions:

$$I(s) = I_{\mathrm{at}}(s) + I_{\mathrm{mol}}(s) \quad \text{(eq. S2)}$$

The atomic scattering term, $I_{\mathrm{at}}(s)$, does not contain structure information and can be calculated easily, provided the empirical formula of the target molecule is known. For a molecule containing N atoms, the atomic scattering term is expressed as the sum of each atomic differential cross-section (each being equal to the modulus squared of the elastic scattering amplitude $f_i(s)$):

$$I_{\mathrm{at}}(s) = \sum_{i=1}^{N} |f_i(s)|^2 \quad \text{(eq. S3)}$$

The scattering amplitudes for a 3.7 MeV incident electron were calculated using the ELSEPA code.[1] The molecular scattering term, $I_{\mathrm{mol}}(s)$, encodes information on the internuclear distances in the target molecules. For an isotropic sample, $I_{\mathrm{mol}}(s)$ is expressed as the sum of interference terms for all possible atom pairs:

$$I_{\mathrm{mol}}(s) = \sum_{i=1}^{N} \sum_{j\neq i}^{N} |f_i(s)||f_j(s)| \frac{sin(sr_{ij})}{sr_{ij}} \quad \text{(eq. S4)}$$

where $f_i(s)$ and $f_j(s)$ are the elastic scattering amplitudes for the ith and the jth atoms, respectively; and r_{ij} is the internuclear distance between the ith and j^{th} atoms.

Diffraction data are typically presented in the form of modified scattering intensities which enhance the oscillations in the I_{mol} term and suppress the rapid drop in scattering intensity as a function of s imparted by the s^{-2} scaling in the elastic scattering amplitude. The modified scattering intensity, $sM(s)$, is defined as:

$$sM(s) = \frac{I_{\mathrm{mol}}(s)}{I_{\mathrm{at}}(s)} s \quad \text{(eq. S5)}$$

A method, developed by Ihee et al.,[2] can be used to calculate experimental modified scattering intensities, $sM_{\mathrm{exp}}(s)$, as the I_{mol} and I_{at} cannot be separated experimentally:

$$sM_{\mathrm{exp}}(s) = \frac{I_{\mathrm{exp}}(s) - I_{\mathrm{bkg}}(s)}{I_{\mathrm{at}}(s)} s \quad \text{(eq. S6)}$$

where $I_{\mathrm{exp}}(s)$ is the experimentally measured scattering intensity and $I_{\mathrm{bkg}}(s)$ is an estimate of the instrument-specific background and atomic scattering contributions. In this work, the $I_{\mathrm{bkg}}(s)$ term was approximated by fitting a sum of exponents to the zero-crossing of the theoretical $I_{\mathrm{mol}}(s)$ term for 2(5H)-thiophenone.

The $sM(s)$ curve can be approximated to a sum of sine waves, arising from all internuclear distances in the target molecule. Therefore, the $sM(s)$ curve can be decomposed into a pair-

distribution function (PDF) of all contributing interatomic distances using the following sine transform:

$$\mathrm{PDF}(r) = \int_0^{s_{\max}} sM(s)sin(sr)e^{-ks^2}ds \qquad \text{(eq. S7)}$$

where $s_{\max}$ is the maximum transfer in the diffraction with adequate signal-to-noise ratio, r is the internuclear distance between atom pairs, and k is a damping factor used to suppress the high s contribution smoothly to zero. A damping factor of 0.03 was used in all static PDF calculations. Prior to the sine transform of $sM_{\exp}(s)$ curves, low-scattering angle data ($s < 0.7$ Å^{-1}) obscured by the hole in the detector was filled in using a linear extrapolation to $s = 0$ in order to minimize the impact of edge artifacts in the experimental PDF.

Generating difference signals from experimental data:

The analysis of time-resolved experimental UED data is based on the difference-diffraction method.[3] This approach suppresses both contributions from molecules not excited by the pump laser and instrument-specific background contributions, thus enhancing the signal arising from photoexcited molecules undergoing structural rearrangements. Our analysis employs a robust representation of the difference-diffraction signal: the fractional change signal, henceforth referred to as $\Delta I/I(s,t)$:

$$\Delta I/I(s,t) = \frac{I(s,t)-I(s,t<0)}{I(s,t<0)} \qquad \text{(eq. S8)}$$

where $I(s,t<0)$ is the reference diffraction signal taken before the arrival of the pump pulse, and $I(s,t)$ is the diffraction intensity recorded at pump-probe delay t. Background contributions unaccounted for by the difference-diffraction method are removed from the experimental difference-signal by the fitting and subtracting of a low-order polynomial from the $\Delta I/I(s,t)$.

The experimental time-dependent difference pair distribution functions, $\Delta\mathrm{PDF}(r,t)$, shown in Fig. 2 of the main text, were calculated by applying the sine-transform of time-dependent difference-modified scattering curves, $\Delta sM_{\exp}(s,t)$:

$$\Delta sM_{\exp}(s,t) = \frac{I(s,t)-I(s,t<0)}{I_{\mathrm{at}}(s)}s \qquad \text{(eq. S9)}$$

$$\Delta\mathrm{PDF}(r,t) = \int_0^{s_{\max}} \Delta sM_{\exp}(s,t)sin(sr)e^{-ks^2}ds \qquad \text{(eq. S10)}$$

A damping factor of 0.03 is used in all PDF and ΔPDF calculations and missing low-scattering angle data was filled in by linear extrapolation to zero.

Detector size to momentum transfer vector calibration:

The conversion between the detector pixel size and the momentum transfer vector, *s*, was calibrated using the known positions of Bragg reflection from a bismuth telluride (Bi_2Te_3) single crystal sample measured at the beginning of the experiment. The value of this conversion was then optimized for each dataset by comparing the theoretical and experimental static (no optical pump) scattering signatures for 2(5H)-thiophenone.

Time-zero determination:

During the experiment, the position of the time-zero was estimated based on the Debye-Waller profile of the diffraction signal of an optically pumped single crystal of silicon. This position was refined during the data analysis process to represent the half-maximum of the absolute percentage change of the difference-diffraction signal.

Error estimation of the experimental signal:

The statistical uncertainty of the experimental signal was estimated using a standard bootstrapping analysis. The UED dataset, which consisted of a pool of 178 unique scans, was randomly resampled with replacement 150 times to produce 150 bootstrapped datasets. Each bootstrapped dataset was analyzed separately, thereby enabling the mean and standard deviation for all relevant analysis outputs to be evaluated. Fig. S2 shows the uncertainty of the UED measurement represented as one standard deviation of the calculated difference-diffraction signal, $\Delta I/I(s,t)$, across the bootstrapped datasets. Note that the estimated uncertainty of the measurement is substantially smaller than the amplitude of the signal across all positive time delays. Saturation of bootstrapped uncertainty reflected in the difference-diffraction standard deviation and standard error was observed after 150 bootstrapped datasets (see Fig. S3).

Assessment of the presence of photoionization in the UED signal:

The plasma lensing effect, first reported by Dantus and Zewail,[4] was used to assess the presence of photoionization in the probed sample volume. Briefly, the plasma field generated by the separation of charges during an ionization event induces a deflection in the incident electron beam.[5] This phenomenon is expressed in the UED signal as a strong persistent difference signal at low-scattering angle ($s < 1$ Å^{-1}) and is accompanied typically by a lensing of the undiffracted electron beam (I_0 signal).[6] The fluence-dependence of the difference signal at $s < 1$ Å^{-1}, depicted in Figure S13A, shows no appreciable change in signal levels at low-scattering angles for pump energies below 25 μJ. Therefore, we conclude that photoionization is unlikely to be present in the 15 μJ dataset discussed in this manuscript. This observation is corroborated further by the lack of electron beam lensing or deflection shown in Figure S13D-F.

Theory

Generation of theoretical scattering signals:

The theoretical static PDF(r) and time-dependent $\Delta I/I(s,t)$ and ΔPDF(r,t) signals in the main text were calculated in accordance to the independent atom model (IAM) [7, 8] using the nuclear configurations captured along the 43 (NA+BO)MD trajectories and eqs. 7, 8 and 10, respectively. The BOMD trajectories were refined with a monotonic time step of 0.5 fs (accounting for the difference in time step: 0.5 fs for NAMD and 0.1 fs for BOMD).

Retrieval of photoproduct relative populations:

The relative populations for the photoproducts were retrieved directly from the UED signal by fitting a linear combination of basis functions selected to reflect the average scattering signatures of photoproducts to the experimental difference-diffraction signal, $\Delta I/I(s,t)$. This approach allowed the relative abundances, *i.e.,* relative populations of 2(5H)-thiophenone, ring-opened and episulfide products to be determined experimentally and their evolution across the experimental time window mapped. The fitting of photoproducts was carried out in reciprocal space as it provides a more robust experimental signal from which quantitative information can be extracted. Although more intuitive, the analysis of real-space signals generated by the sine-transform of difference-diffraction signals requires the implementation of artifact mitigation strategies, such as the filling of missing data at low-scattering angles and the damping of high-scattering angle contributions, both of which impact the breath and amplitude of the calculated ΔPDF. For this reason, the real-space features are considered to offer a less quantitative signal from which to determine information regarding relative abundances of photoproducts.

Basis function selection:

The three photoproduct basis functions used to fit the experimental difference-diffraction signal were obtained by averaging the theoretical $\Delta I/I(s,t)$ signals for 2(5H)-thiophenone, ring-opened and episulfide products in the range $1 \leq t \leq 2$ ps. The temporal evolutions of the two strongest scattering features in the theoretical $\Delta I/I(s,t)$ functions for these three photoproducts, depicted in Figs. S6B and S6C, plateau after ~ 1 ps. This plateau reflects the settling of photoproduct geometries into discrete ensembles with a breadth of conformations that adequately represents each photoproduct classification. For this reason, the average signal in the range $1 \leq t \leq 2$ ps was selected as an adequate time-independent basis function with which to fit the experimental signal. However, a consequence of using time-independent basis functions based on the average scattering signals of vibrationally hot photoproducts in the ground state is that they are unlikely to optimally capture all structural changes taking place in the early times following photoexcitation, when the molecule is in an excited electronic state.

Fitting routine:

A global search algorithm implemented in Matlab was used to minimize the root-mean-square error (RMSE) between the theoretical $\Delta I/I(s,t)$ signal calculated from a linear combination of photoproduct basis functions and the experimental $\Delta I/I(s,t)$ signal for each time delay visited in the UED experiment. The fits were performed on the raw experimental signal without the application of high or low pass filters. This avoids the biasing of the fit by an arbitrary selection of high and/or low pass cutoff frequency. During the fitting routine, the theoretical signal is scaled to reflect the percentage of molecules in the experimentally probed volume that were optically excited by the UV pulse and therefore contribute to the experimental difference-diffraction signal. This scaling factor, which is constant across the entire dataset, was determined by fitting the average experimental signal between 1 and 2 ps to a scaled linear combination of basis functions. The results of this fit, which are shown in the bottom panel of Fig. 3D, indicate that 3% of the molecules in the probed volume are photoexcited (scaling factor of 0.03). To remove biases introduced by the selection of the momentum transfer range included in the fit, fitting routines were repeated 8 times with randomized starting and end points between 0.65-2 and 7-10 Å, respectively. Moreover, the fitting of both the average experimental signal between 1 and 2 ps and the fitting of the experimental signal for unique time delays was repeated for each of the 150 bootstrapped datasets. The reported relative population uncertainties are, therefore, a reflection of both the variance of results across different momentum transfer range selections and the inherent variability of bootstrapped datasets. The time-evolving electron diffraction signals reported here could, in principle, surely be reproduced by several combinations of bond distances/angles. It is important to stress that the present interpretation is specifically based on the results of *ab initio* nonadiabatic and adiabatic molecular dynamics simulations of the excited- and ground-state dynamics of 2(5H)-thiophenone following photoexcitation at 266 nm. The exact same simulations were used to interpret and reproduce the results from TRPES studies of 2(5H)-thiophenone following excitation under very similar conditions.[9]

Benchmarking of the relative population retrieval methodology:

The performance of the quantitative retrieval of photoproduct relative populations was assessed by applying the fitting routine described above to the total theoretical difference-diffraction signal. The results of this benchmark fit show that the proposed methodology is able to retrieve the relative populations of the three photoproducts identified by classification II. Good quantitative agreement was found between the true relative populations obtained from the (NA+BO)MD simulations (fraction of trajectories showing a given photoproduct at a given time), depicted in Fig. S7A, and those retrieved by fitting the temporally convolved theoretical UED signal, shown in Fig. S7B. As discussed above, the basis functions appear to map less well to the structural rearrangements observed at early pump-probe delays, when the molecule is in an excited electronic state, as seen

by the slight discrepancies observed between true theoretical and retrieved relative populations obtained for the 0 – 350 fs time window.

The impact of basis function selection on the goodness of fit was investigated by inspecting the RMSE obtained for a series of fits using different combinations of three photoproduct basis functions. For added flexibility, classification I was used to generate the three photoproduct basis functions, as it yields two additional basis functions corresponding to the ring-opened photoproducts P1 and P2 (the nomenclature for the photoproduct is defined in Figure S4). Figure S8, which depicts the fit of the average experimental signal between 1 and 2 ps using three different combinations of basis functions, shows that the inclusion of a basis function for episulfide results in a better overall fit and allows the close reproduction of the experimental signal in the $1 < s < 4$ $Å^{-1}$ region. It is important to note that this region of momentum transfer range offers the best signal to noise ratio and therefore can be used to assess the impact of basis function selection on the goodness of fit. We also emphasize that no high band pass filter is applied to the raw data to avoid arbitrarily selecting a cut-off and the potential artifacts that such a procedure can induce. The results of these fits, which are summarized in Table S1, show that the inclusion of an episulfide basis function substantially improves the goodness of fit, *i.e.*, lowers the fit RMSE below $10 \cdot 10^{-4}$, for all combinations of basis functions tested. Therefore, episulfide geometries are crucial to the appropriate modeling of scattering signals which arise from the ensemble of photoproducts produced during the UED experiment. Moreover, the results of fits carried out using 3 and 4 photoproduct basis functions, summarized in Table S2, show that the episulfide relative population is insensitive to the exact number and nature of any additional basis functions included in the fit. Therefore, the relative population for episulfide does not act as a sink of residuals in the fitting routines but is a robust fitting parameter.

Supporting Figures and Tables

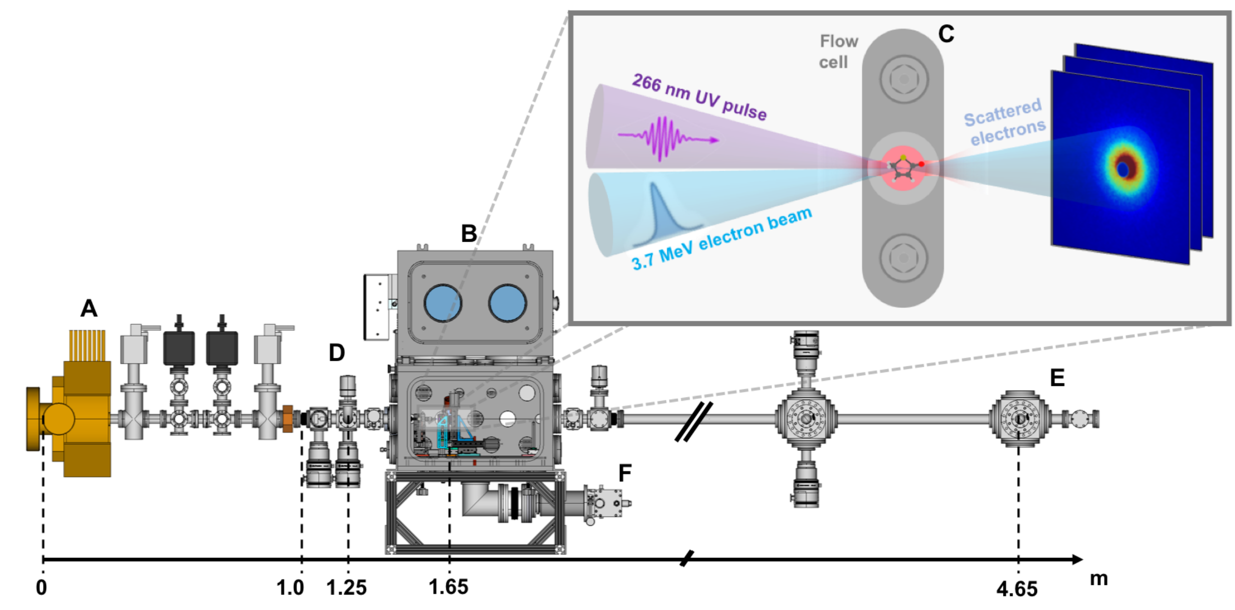

Fig. S1: Schematic of experimental setup. At the SLAC National Accelerator Laboratory UED instrument, 3.7 MeV electron bunches generated by a photocathode radio frequency electron gun (A) are accelerated towards an experimental chamber (B) where they impinge upon a volume of 2(5H)-thiophenone gas inside a flow cell (C). The sample volume is optically pumped by a UV laser pulse coupled into the experimental chamber by an in-vacuum holey mirror (D). The scattered electrons are collected 3 meters downstream of the pump-probe overlap region by a scintillator coupled CCD detector (E). Exhausted 2(5H)-thiophenone gas is condensed in a cryogenically cooled high surface area cold trap (F). The inset panel shows a schematic representation of the UED interaction volume and resulting diffraction pattern.

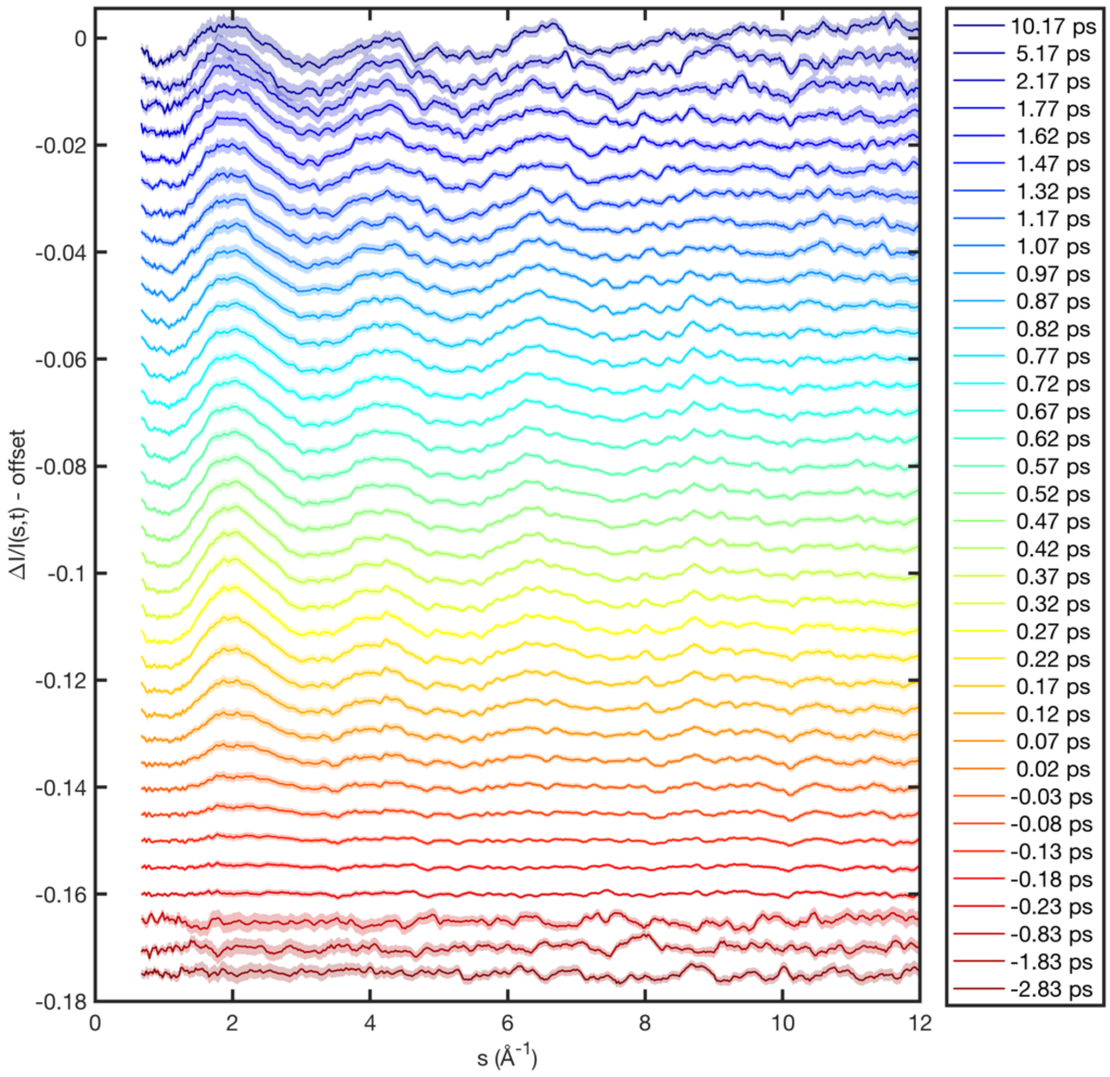

Fig. S2: Experimental signal uncertainty. Experimental difference-diffraction signal, $\Delta I/I(s,t)$, for all 37 time delays visited in the UED experiment. Positive and negative delays represent, respectively, the UV pump pulse arriving before and after the UED probe pulse. The shaded areas reflect one standard deviation across the 150 bootstrapped datasets.

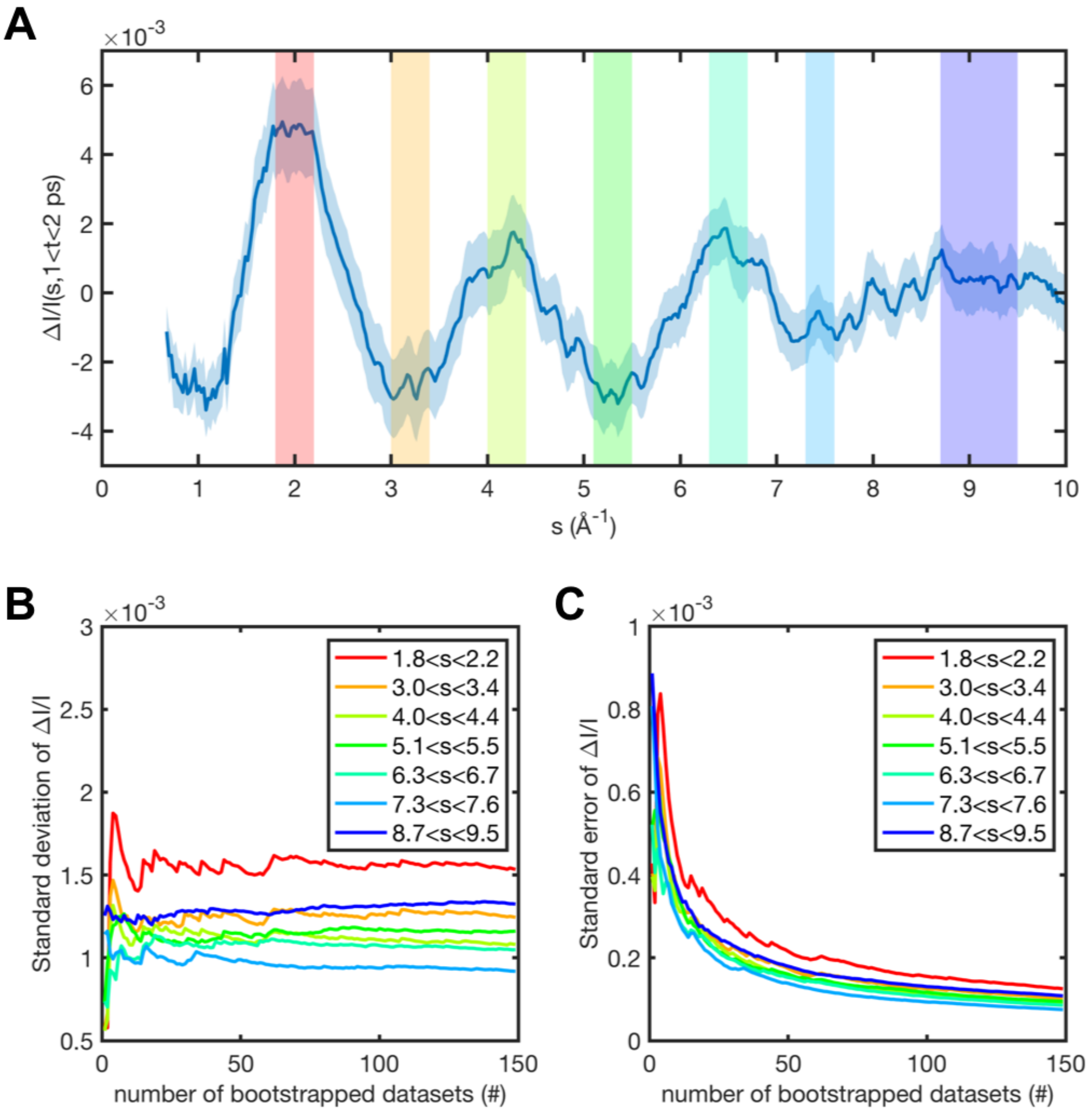

Fig. S3: Uncertainty development through the dataset bootstrapping. (**A**) shows the average experimental difference-diffraction signal for the time interval between 1 and 2 ps, with the shaded area representing one standard deviation across the 150 bootstrapped datasets. The shaded vertical bands represent the scattering features used to monitor the development of the uncertainty as a function of the number of bootstrapped datasets. (**B**) and (**C**) show, respectively, the development and saturation of the standard deviation and standard error of the difference-diffraction signal as a function of the number of bootstrapped datasets used in the analysis.

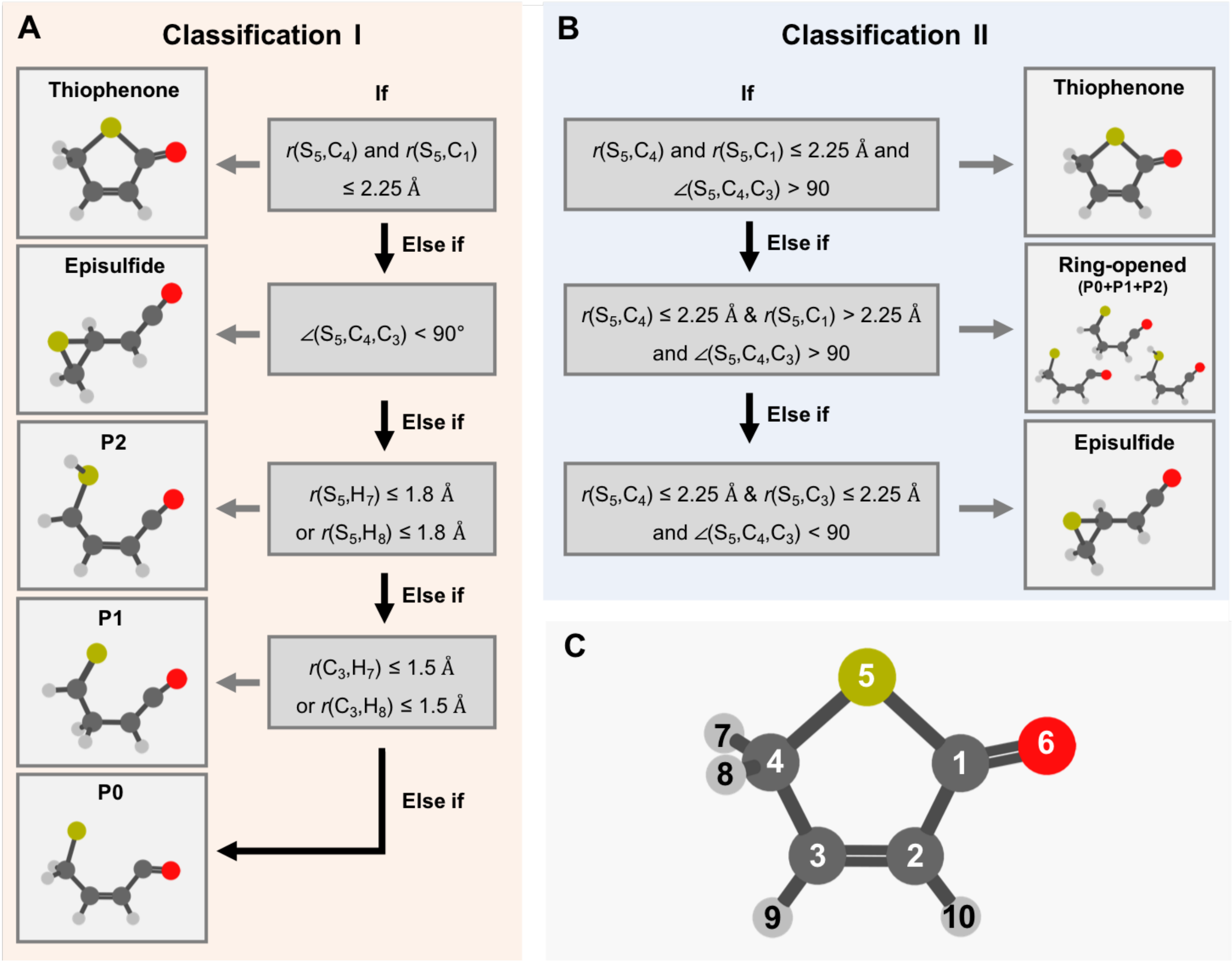

Fig. S4: Decision trees for the classification of photoproducts. (**A**): decision tree for classifying all photoproducts identified by our (NA+BO)MD simulations and reported in Ref. 11. (**B**): decision tree for the classification of photoproducts whose scattering signatures are distinct enough that their relative contributions can be unambiguously assigned using the experimental UED signal.

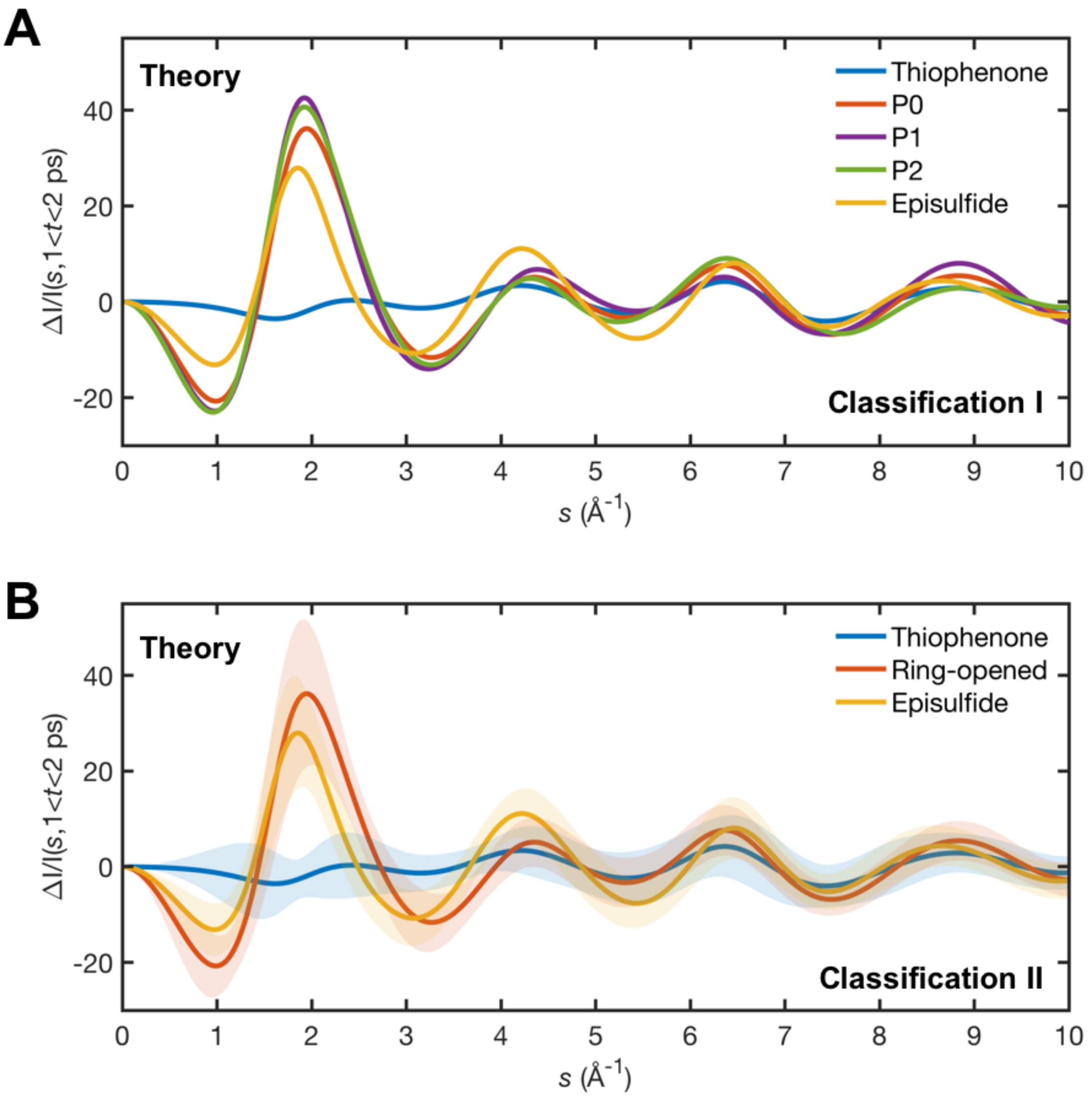

Fig. S5: Photoproduct signatures according to classifications I and II. (**A**): average scattering signatures of five photoproducts identified according to classification I (see Fig. S4). The scattering signatures of the ring-opened photoproducts P0, P1 and P2 are nearly indistinguishable (see Fig. S4 for the nomenclature of the photoproducts). Therefore, under classification II shown in (**B**), the relative scattering contributions from P0, P1 and P2 are grouped under a single category called *ring-opened*. The shaded regions in (**B**) represent one standard deviation across the ensemble of classified geometries. An equivalent representation is omitted from (**A**) for clarity purposes.

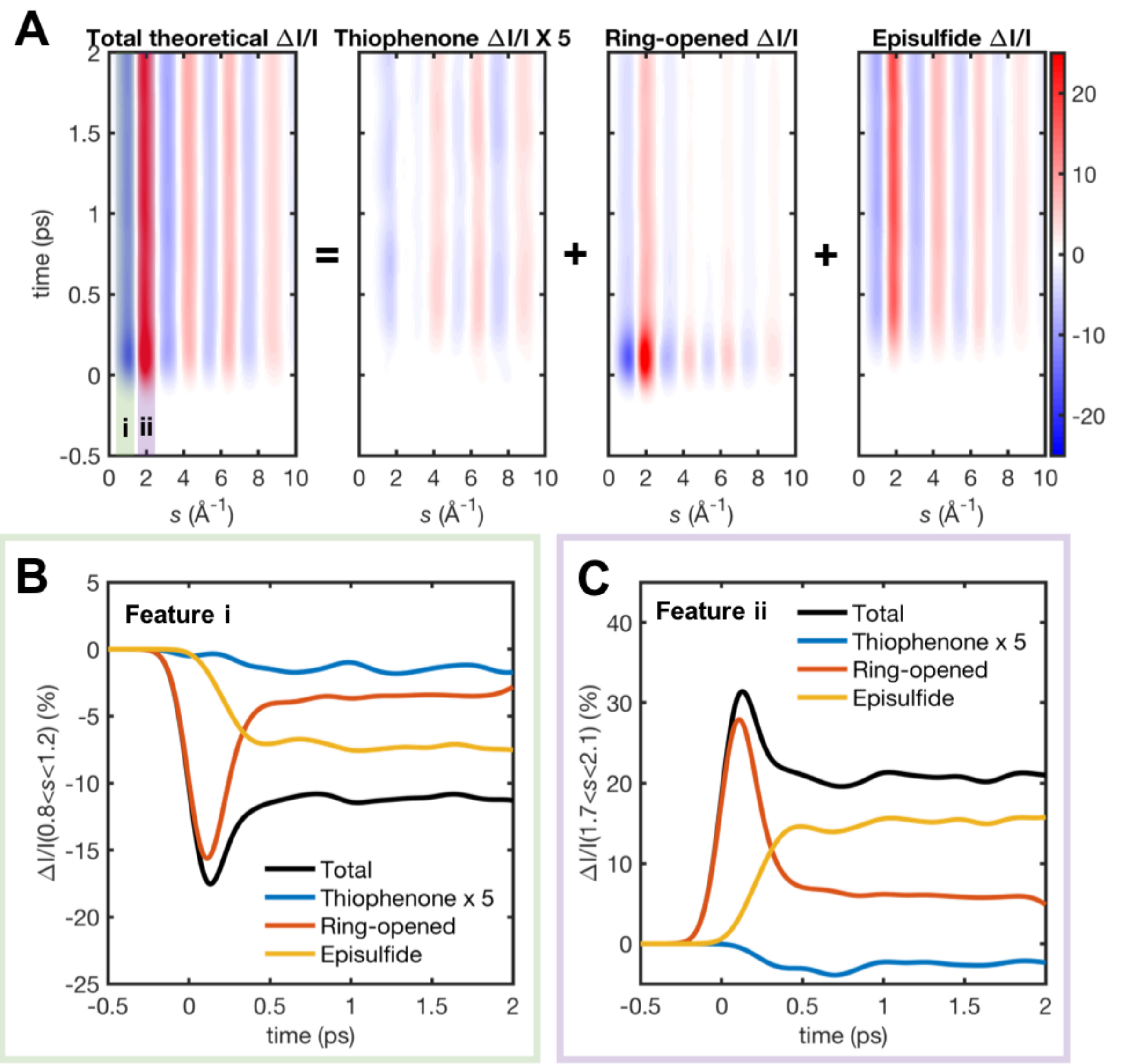

Fig. S6: Photoproduct basis function selection. (**A**): theoretical total $\Delta I/I(s,t)$ signal and relative contributions from each of the three photoproducts identified using classification II. These theoretical $\Delta I/I(s,t)$ maps have been convolved with a 230-fs FWHM Gaussian function which serves as an estimate for the instrument response function. (**B**) and (**C**): temporal evolution of the two strongest scattering features centered around: i) $+0.8 < s < +1.2$ and ii) $+1.7 < s < +2.1$ Å^{-1}, respectively. The temporal evolution of the total theoretical scattering signals and the relative contributions of each photoproduct reach a plateau after ~1 ps. The lack of large amplitude modulations on the temporal evolution of the photoproduct contributions to the total scattering signal at $t > 1$ ps enabled the use of time-independent photoproducts basis functions based on the average scattering signal in the range $+1 \leq t \leq +2$ ps.

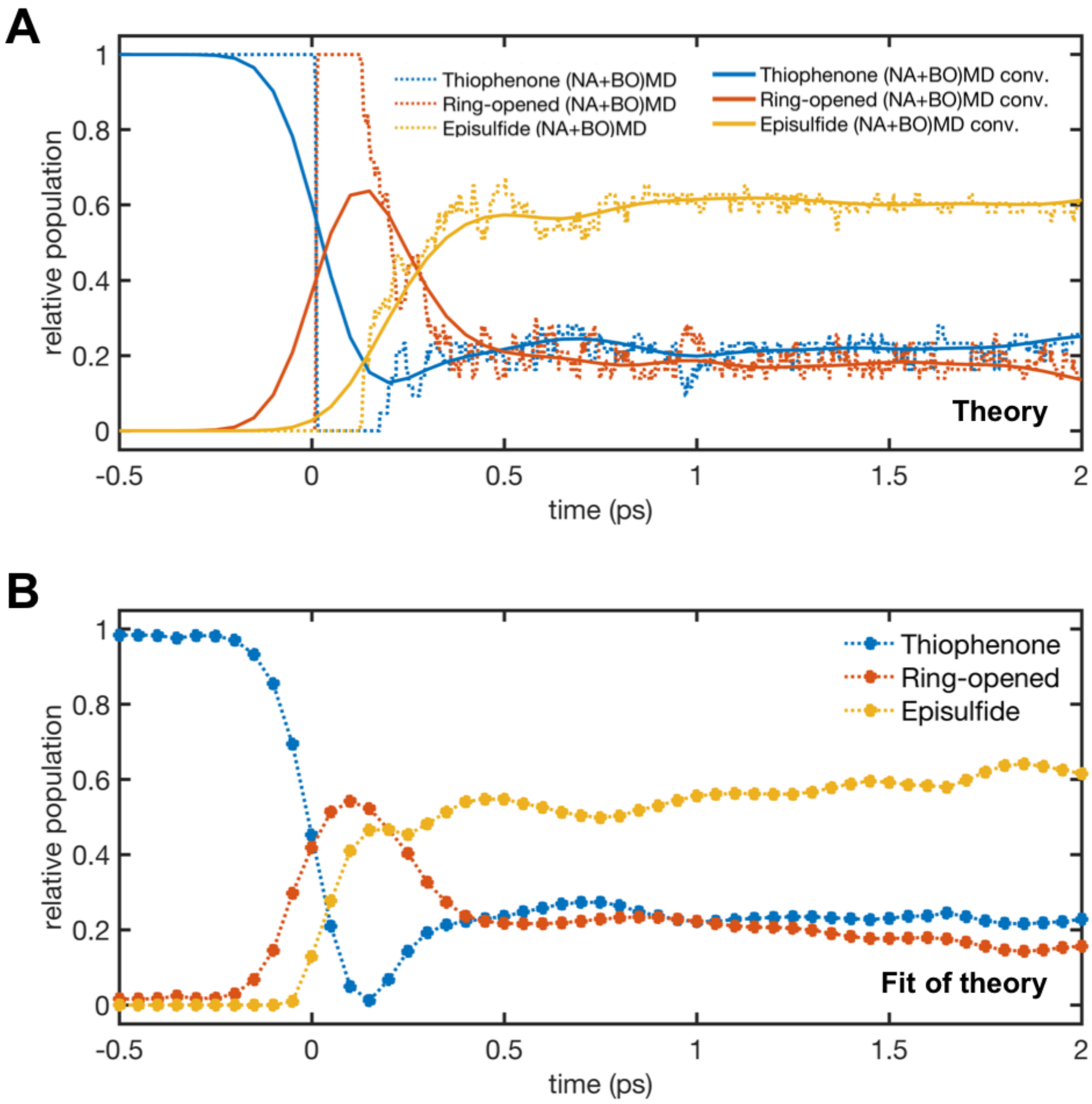

Fig. S7: Benchmarking of the photoproduct relative population retrieval. (**A**): temporal evolution of the photoproduct relative populations obtained from the (NA+BO)MD simulations directly (dashed lines). Each relative population in this case is defined as the fraction of trajectories, at a given time, entering a particular category identified according to classification II. The solid lines in (**A**) were obtained by convolving the time-dependent photoproduct relative populations with a 230-fs FWHM Gaussian function which approximates the experimental IRF. (**B**): temporal evolution of the photoproduct relative populations obtained by fitting the temporally convolved total theoretical signal, $\Delta I/I(s,t)$, with the time-independent basis functions shown in Fig. S5B.

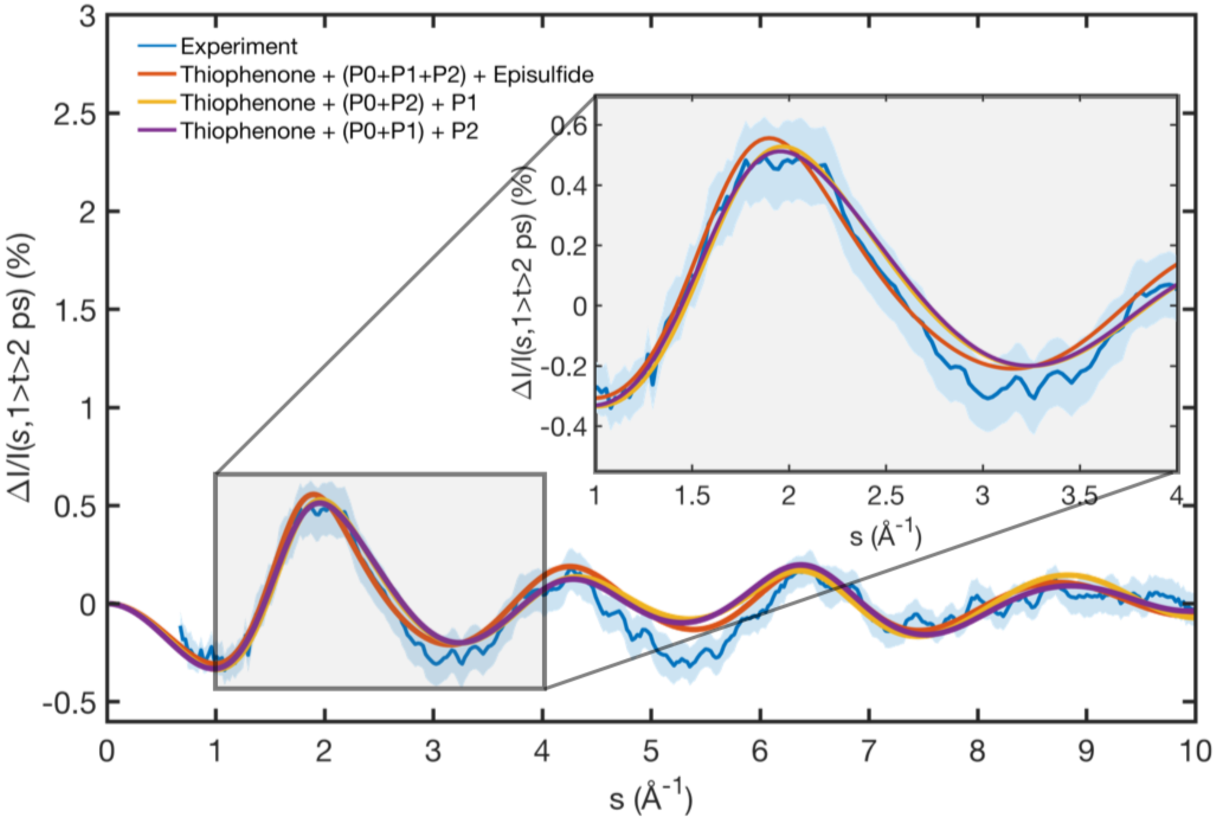

Fig. S8: Benchmarking of basis function selection. Fitting of the average experimental signal between 1 and 2 ps with three sets of basis functions including and excluding episulfide. The inset shows a zoom of the $1 < s < 4$ Å^{-1} region. See Fig. S4 for the nomenclature of the photoproducts.

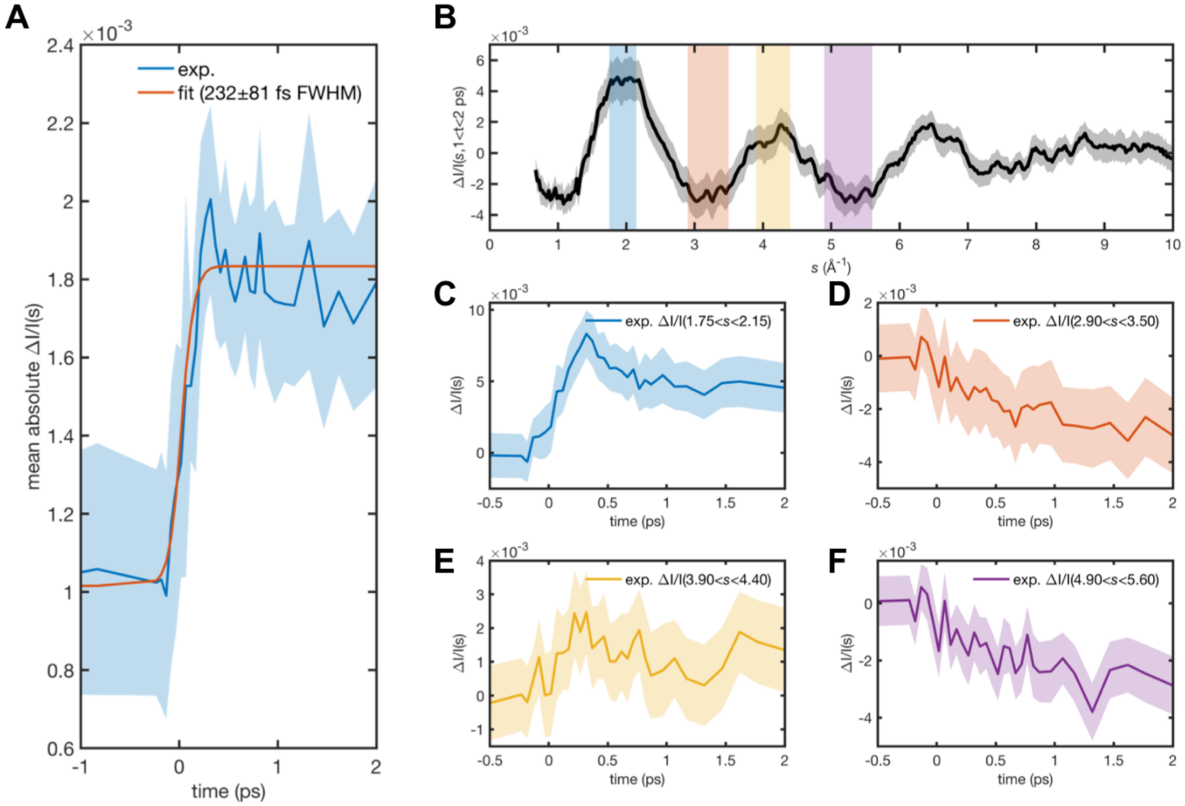

Fig. S9: Experimental signal line-outs. (A) shows the experimental average absolute difference-diffraction signal as function of time delay and corresponding Gaussian error fit which is used to estimate the instrument response function of the UED instrument. (**B**) shows the average experimental difference-diffraction signal for the time interval between 1 and 2 ps, with the shaded areas representing the four strongest features used to produce the line-outs in panels **(C)** to **(F)**. The shaded areas in panels **(C)** to **(F)** represent one standard deviation across the 150 bootstrapped datasets.

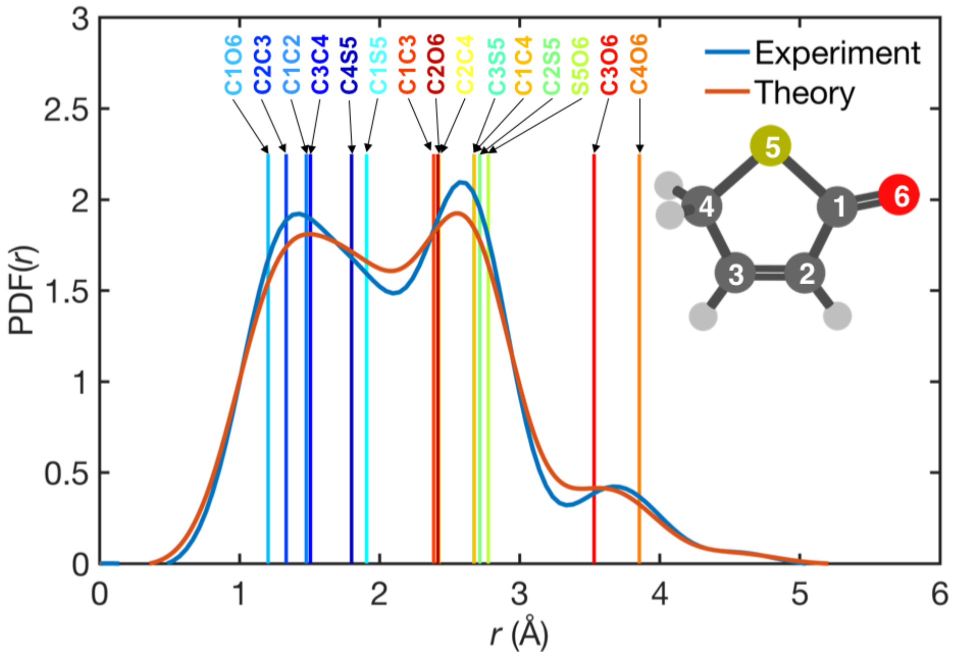

Fig. S10: Assignment of the steady-state PDF for 2(5H)-thiophenone. The vertical sticks represent average interatomic distances for heavy atoms obtained from the geometries sampled from the Wigner distribution.

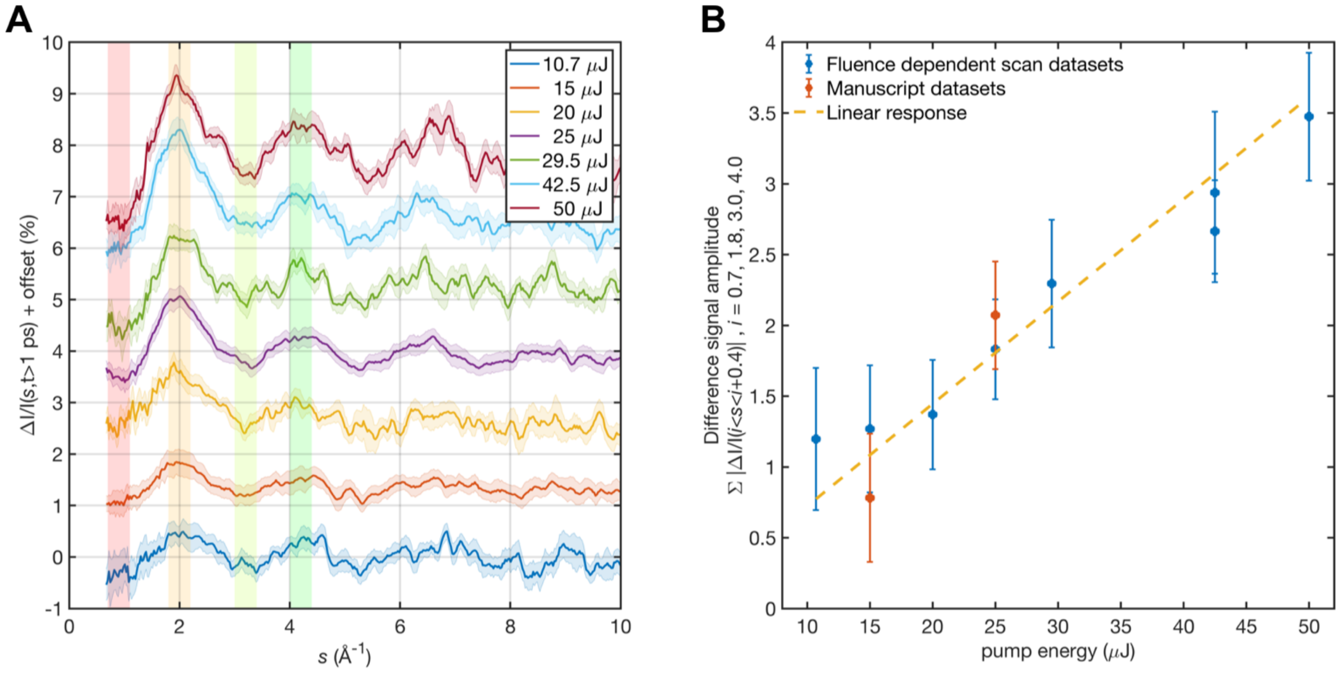

Fig. S11: Fluence-dependence plot. The shaded vertical bands in panel **A** represent the scattering features used to calculate the difference signal amplitude used in the fluence-dependence plot in panel **B**. Panel **B**: The red dot at 15 μJ corresponds to the dataset presented in the main text and the Supplementary Materials, and the red dot at 25 μJ is for the additional dataset analyzed in Fig. S12. We also note that the pump conditions in the present experiment were comparable to those in our prior photoelectron spectroscopy experiment on 2(5H)-thiophenone performed at FERMI (*10*), i.e., comparable UV pulse duration and focus size (200 μm at FERMI as opposed to 230 μm for MeV-UED) and slightly higher pulse energy (25 μJ at FERMI as compared to 15 μJ for the UED data presented in the main text). Since the FERMI experiment detected both photoelectrons and ions, it was possible to directly monitor the ionization of the target molecules by the pump pulse and to unambiguously determine that the degree of ionization under these conditions was negligible. The pump pulse energy of 25 μJ for the FERMI experiment was chosen because it was right below the onset of any noticeable ionization signal.

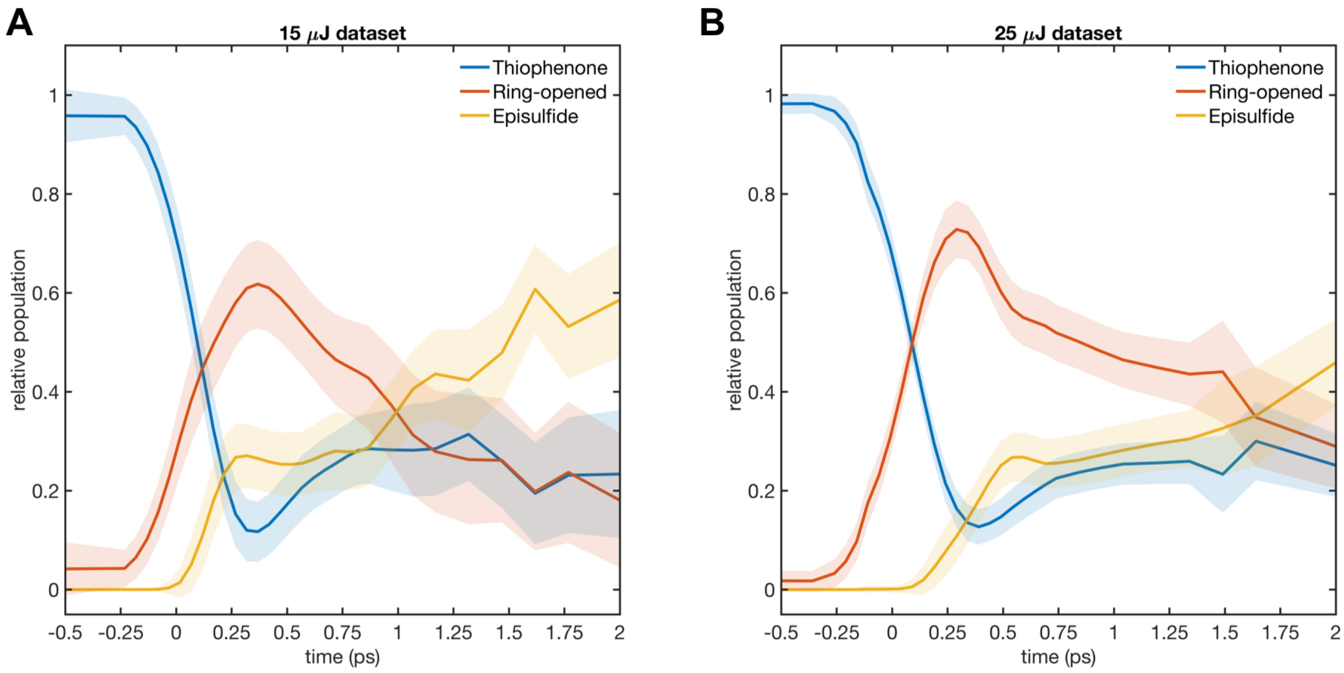

Fig. S12: Time-resolved relative populations for the three families of photoproducts. Time-dependent photoproduct populations extracted from datasets obtained with pump energies of 15 μJ (panel **A**, same as Fig. 5B in the main text) and 25 μJ (panel **B**). The two datasets shown here correspond to the two red dots in the panel **B** of Fig. S11.

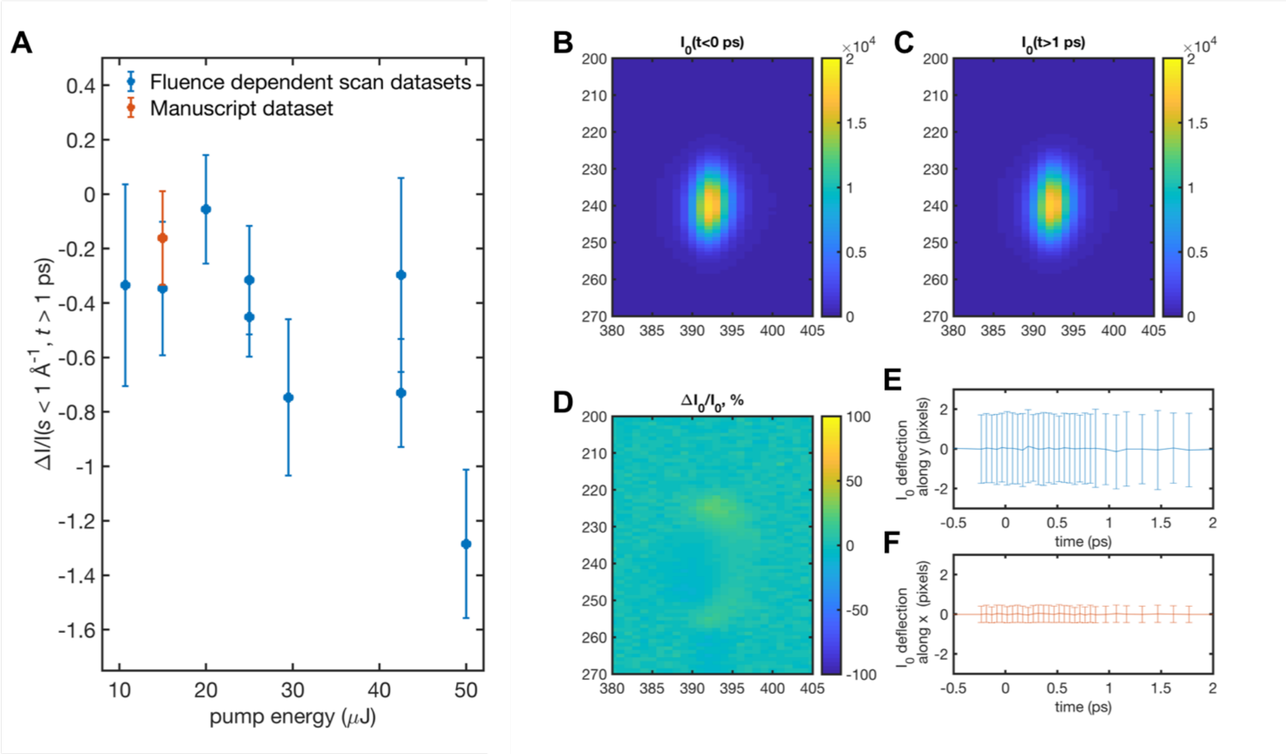

Fig. S13: Assessment of the presence of photoionization induced contributions to the UED signal. (**A**) shows a plot of the average difference signal at $s < 1$ Å^{-1} and $t > 1$ picosecond as a function of pump energy. The red dot at 15 μJ corresponds to the dataset presented in the main text. (**B**) and (**C**) are spatially resolved false-color plots showing the average intensity of the undiffracted electron beam (I_0) before time-zero and after 1 picosecond, respectively. The intensity, shape and position of the undiffracted electron beam were measured on a 2-dimensional detector (I_0 detector) located immediately downstream of the main diffraction detector. The I_0 intensity is defined in detector counts by the false-color scale to the immediate right of panels B and C. The percentage difference signal between panels B and C is depicted in panel (**D**) and shows no appreciable lensing of the undiffracted electron beam. (**E**) and (**F**) show the position of the undiffracted electron beam centre-of-mass as a function of time along the x and y directions, respectively. We note that these data shown no time-dependent deflection of the undiffracted electron beam.

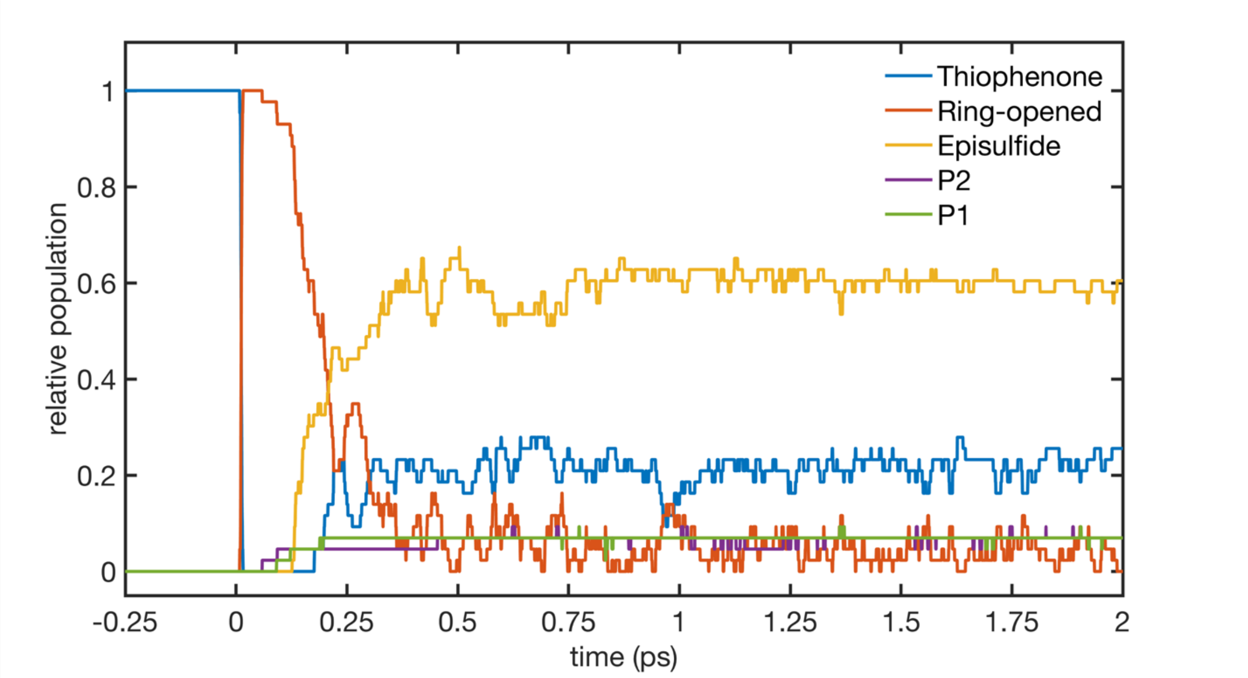

Fig. S14: Time-resolved relative populations for the different photoproducts. Temporal evolution of the photoproduct relative populations obtained from the (NA+BO)MD simulations, with the different ring-opened photoproducts – ring-opened, P1 and P2 – presented separately (see Fig. S4 for notation).

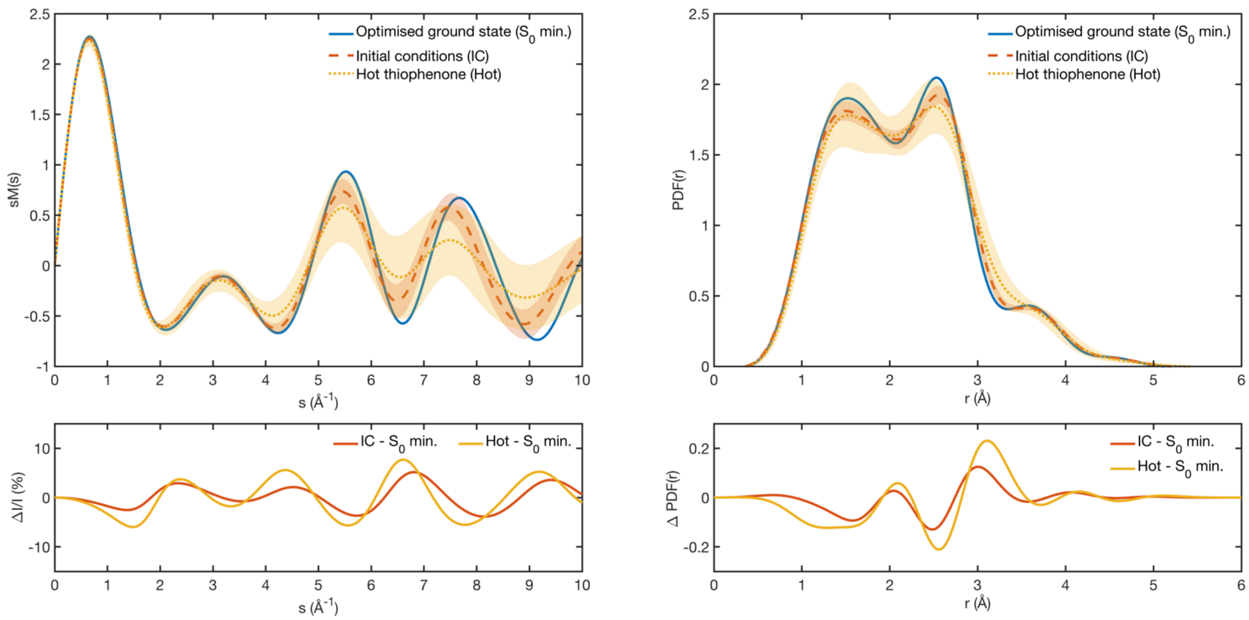

Fig. S15: Simulated steady-state UED signals for 2(5H)-thiophenone from different representative structures. Steady-state $sM(s)$ (left) and PDF (right) for 2(5H)-thiophenone calculated from the ground-state optimized geometry of the molecule (S_0 min), the distribution of initial conditions sampled from a Wigner distribution mimicking the ground-state probability density at 0K (IC), and the distribution of 2(5H)-thiophenone geometries obtained from the (NA+BO)MD dynamics and representative of the ground-state athermal dynamics of (reformed) 2(5H)-thiophenone following the nonradiative decay (Hot). The lower panels show the $\Delta I/I$ and the ΔPDF obtained by subtracting the S_0 min signal.

Table S1. Impact of basis function selection on the goodness of fit.

Table showing the root mean square error for fits of the average experimental signal between 1 and 2 ps using different combinations of three photoproduct basis functions (see Fig. S4 for the nomenclature of the photoproducts).

**Basis Function**	**RMSE x 10^{-4}**
2(5H)-thiophenone + (P0+P1+P2) + Episulfide	9.9±2.9
2(5H)-thiophenone + (P0+P2) + P1	11.0±2.7
2(5H)-thiophenone + (P0+P1) + P2	10.7±2.9
Episulfide + (P0+P2) + P1	9.8±3.0
Episulfide + (P0+P1) + P2	9.9±2.9
P0 + P2 + P1	11.5±2.9

Table S2. Impact of basis function selection and number on fit results.

Table showing the relative populations, excitation percentage (exc.), and RMSE retrieved from fits of the average experimental signal between 1 and 2 ps using different combinations of photoproduct basis functions (see Fig. S4 for the nomenclature of the photoproducts).

**Basis functions**	**2(5H)-thiophenone (%)**	**Ring-opened (%)**	**P2 (%)**	**P1 (%)**	**Episulfide (%)**	**exc. (%)**	**RMSE x 10^{-4}**
2(5H)-thiophenone + (P0+P1+P2) + Episulfide	24±15	26±11	-	-	50±13	2.6±0.4	9.9±2.9
2(5H)-thiophenone + (P0+P2) + P1 + Episulfide	19±15	29±12	-	0±1	51±12	2.5±0.4	9.6±2.9
2(5H)-thiophenone + (P0+P1) + P2 + Episulfide	29±15	2±5	24±11	-	46±14	2.6±0.4	9.7±2.9

References

1. Francesc, S.; Aleksander, J.; Cedric, P., ELSEPA - Dirac Partial-Wave Calculation of Elastic Scattering of Electrons and Positrons by Atoms, Positive Ions and Molecules. **2005**.
2. Ihee, H.; Lobastov, V. A.; Gomez, U. M.; Goodson, B. M.; Srinivasan, R.; Ruan, C. Y.; Zewail, A. H., Direct imaging of transient molecular structures with ultrafast diffraction. *Science* **2001,** *291* (5503), 458-62.
3. Ihee, H.; Cao, J.; Zewail, A. H., Ultrafast electron diffraction: structures in dissociation dynamics of $Fe(CO)_5$. *Chem. Phys. Lett.* **1997,** *281* (1), 10-19.
4. Dantus, M.; Kim, S. B.; Williamson, J. C.; Zewail, A. H., Ultrafast Electron-Diffraction .5. Experimental Time Resolution and Applications. *J Phys Chem-Us* **1994,** *98* (11), 2782-2796.
5. Centurion, M.; Reckenthaeler, P.; Trushin, S. A.; Krausz, F.; Fill, E. E., Picosecond electron deflectometry of optical-field ionized plasmas. *Nat Photonics* **2008,** *2* (5), 315-318.
6. Yang, J.; Zhu, X.; F Nunes, J. P.; Yu, J. K.; Parrish, R. M.; Wolf, T. J. A.; Centurion, M.; Gühr, M.; Li, R.; Liu, Y.; Moore, B.; Niebuhr, M.; Park, S.; Shen, X.; Weathersby, S.; Weinacht, T.; Martinez, T. J.; Wang, X., Simultaneous observation of nuclear and electronic dynamics by ultrafast electron diffraction. *Science* **2020,** *368* (6493), 885-889.
7. McMorrow, D.; Als-Nielsen, J., *Elements of modern X-ray physics*. John Wiley & Sons: 2011.
8. Parrish, R. M.; Martínez, T. J., Ab Initio Computation of Rotationally-Averaged Pump–Probe X-ray and Electron Diffraction Signals. *J. Chem. Theory Comput.* **2019,** *15* (3), 1523-1537.
9. Pathak, S.; Ibele, L. M.; Boll, R.; Callegari, C.; Demidovich, A.; Erk, B.; Feifel, R.; Forbes, R.; Di Fraia, M.; Giannessi, L.; Hansen, C. S.; Holland, D. M. P.; Ingle, R. A.; Mason, R.; Plekan, O.; Prince, K. C.; Rouzée, A.; Squibb, R. J.; Tross, J.; Ashfold, M. N. R.; Curchod, B. F. E.; Rolles, D., Tracking the ultraviolet-induced photochemistry of thiophenone during and after ultrafast ring opening. *Nat. Chem.* **2020,** *12* (9), 795-800.
